# Synthesis and reactivity of a terminal uranium(iv) sulfide supported by siloxide ligands†
†Electronic supplementary information (ESI) available: Full computational details, ^1^H NMR spectra, and detailed X-ray crystallographic data in CIF format. CCDC 1447982–1447990. For ESI and crystallographic data in CIF or other electronic format see DOI: 10.1039/c6sc00675b


**DOI:** 10.1039/c6sc00675b

**Published:** 2016-05-10

**Authors:** Julie Andrez, Jacques Pécaut, Rosario Scopelliti, Christos E. Kefalidis, Laurent Maron, Michael W. Rosenzweig, Karsten Meyer, Marinella Mazzanti

**Affiliations:** a Institut des Sciences et Ingénierie Chimiques Ecole Polytechnique Fédérale de Lausanne (EPFL) , 1015 Lausanne , Switzerland . Email: marinella.mazzanti@epfl.ch; b Univ. Grenoble Alpes , INAC-SyMMES , RICC , F-38000 Grenoble , France; c CEA , INAC-SyMMES , F-38000 Grenoble , France; d Université de Toulouse et CNRS INSA , UPS , CNRS , UMR 5215 , LPCNO , 135 avenue de Rangueil , 31077 Toulouse , France; e Department of Chemistry and Pharmacy , Inorganic Chemistry , Friedrich-Alexander University Erlangen-Nürnberg , Egerlandstraße 1 , 91058 Erlangen , Germany

## Abstract

The reactions of the tetrasiloxide U(iii) complexes [U(OSi(O^*t*^Bu)_3_)_4_K] and [U(OSi(O^*t*^Bu)_3_)_4_][K18c6] with 0.5 equiv. of triphenylphosphine sulfide led to reductive S-transfer reactions, affording the U(iv) sulfide complexes [SU(OSi(O^*t*^Bu)_3_)_4_K_2_]_2_, **1**, and [{SU(OSi(O^*t*^Bu)_3_)_4_K_2_}_2_(μ-18c6)], **2**, with concomitant formation of the U(iv) complex [U(OSi(O^*t*^Bu)_3_)_4_]. Addition of 1 equiv. of 2.2.2-cryptand to complex **1** resulted in the isolation of a terminal sulfide complex, [SU(OSi(O^*t*^Bu)_3_)_4_K][Kcryptand], **3**. The crucial role of the K^+^ Lewis acid in these reductive sulfur transfer reactions was confirmed, since the formation of complex **3** from the reaction of the U(iii) complex [U(OSi(O^*t*^Bu)_3_)_4_][Kcryptand] and 0.5 equiv. of PPh_3_S was not possible. Reactivity studies of the U(iv) sulfide complexes showed that the sulfide is easily transferred to CO_2_ and CS_2_ to afford S-functionalized products. Moreover, we have found that the sulfide provides a convenient precursor for the synthesis of the corresponding U(iv) hydrosulfide, {[(SH)U(OSi(O^*t*^Bu)_3_)_4_][K18c6]}, **5**, after protonation with PyHCl. Finally, DFT calculations were performed to investigate the nature of the U–S bond in complexes **1**, **3** and **5**. Based on various analyses, triple-bond character was suggested for the U–S bond in complexes **1** and **3**, while double-bond character was determined for the U–SH bond in complex **5**.

## Abstract 2

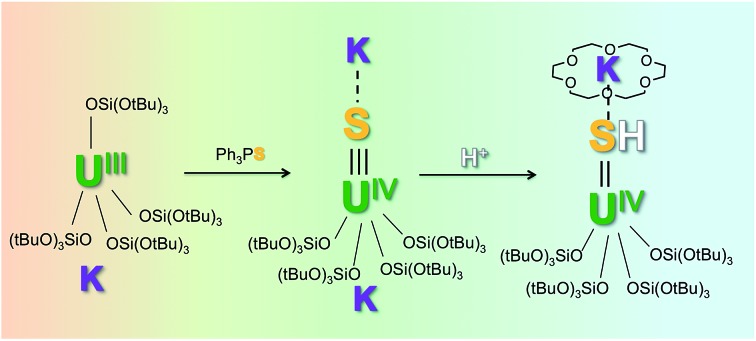
The S-transfer reaction from Ph_3_PS to the tetrasiloxide U(iii) complex [U(OSi(O^*t*^Bu)_3_)_4_K] affords a stable U(iv) triply bonded terminal sulfide that can be protonated to yield a U(iv) doubly bonded terminal hydrosulfide.

## Introduction

Actinide complexes containing terminal oxide, sulfide or nitride ligands involved in multiple bonding with actinide ions are of high current interest, due to both their electronic structures and potential applications in atom transfer chemistry and catalysis.^1^–^11^ Though still considerably less numerous than complexes containing bridging oxo or uranyl (UO_2_^*n*+^) groups, the number of well-characterized terminal uranium oxo complexes has been rapidly increasing in recent years.^3^,^9^,^12^–^16^ In contrast, the majority of attempts to prepare terminal sulfides have resulted in the formation of di-uranium sulfide-bridged complexes^17^–^22^ or mononuclear disulfide complexes^18^,^23^ because of the nucleophilic character of the terminal sulfide. Such nucleophilic character has been demonstrated by reactivity studies of a bridging sulfide with heteroallenes, leading to thiocarbonates.^24^,^25^

Chalcogenide complexes of actinides are also attracting increasing interest because of their importance in energy-related research. Additionally, the nature of the bonding between hard actinides and soft donor atoms and especially the involvement of f-orbitals in these bonds are of great relevance for academia and industry.^26^,^27^ Notably, the efficiency of sulfur-containing ligands in the selective extraction of actinides from spent nuclear fuel^28^ has been attributed to the presence of covalent An–S interactions, which remains a source of debate.^29^–^34^ However, to date, only one ligand system has been reported that enabled the isolation of a terminal uranium sulfide.^16^,^35^ Hayton and co-workers succeeded in synthesizing the terminal U(iv) sulfide complex [US(NR_3_)_3_] (R = SiMe_3_) by disfavouring the formation of bridging sulfide complexes with an ylide capping group during sulfur transfer from S_8_.^16^ The same complex was also prepared by cleavage of a trityl protecting group.^35^ A sodium-capped uranium mononuclear sulfide was also reported (with Cp* as a supporting ligand) that was isolated from the reduction of the thiolate [Cp*_2_U(S^*t*^Bu)_2_] with Na/Hg amalgam.^36^ While bulky ligands are usually used in transition metal chemistry to prevent the formation of bridging oxides and sulfides, this approach has not been successful so far in the preparation of a terminal uranium sulfide complex.

Recently, our group reported the synthesis of a terminal U(v) oxo complex, [UO(OSi(O^*t*^Bu)_3_)_4_K], *via* cooperative two-electron reduction of carbon dioxide by the bulky heterobimetallic uranium(iii) complex [U(OSi(O^*t*^Bu)_3_)_4_K].^14^ Herein, we report the stabilization of a terminal uranium sulfide species by the bulky ligand environment created by four siloxide groups. Moreover, we found that sulfur transfer from the two-electron oxidizing agent Ph_3_PS to the highly hindered uranium centre is favoured by the presence of the Lewis acid K^+^. The reactivity of this U(iv) sulfide with different substrates was also investigated. Additionally, the bonding analysis of the terminal sulfide complex and of the potassium-bound sulfide complex revealed triple-bond character. Most importantly, calculations highlight that the participation of the f-orbitals in the bonding is indeed low.

## Results and discussion

### Syntheses and molecular structures of uranium(iv) sulfides

**Fig. 1:**
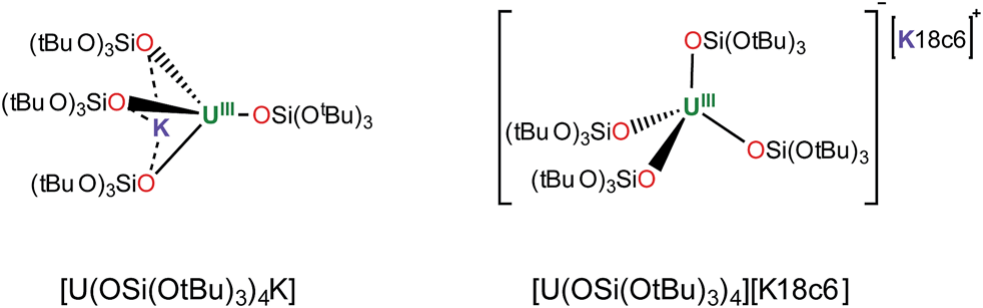
Drawing of complexes [U(OSi(O^*t*^Bu)_3_)_4_K] (left) and [U(OSi(O^*t*^Bu)_3_)_4_][K18c6] (right).

With the goal of preparing a terminal sulfide complex, the bulky U(iii) complex [U(OSi(O^*t*^Bu)_3_)_4_K] (Fig. 1, left) was reacted with elemental sulfur. This reaction led to multiple oxidation products, regardless of the applied stoichiometry (0.125 eq. or 0.25 eq. of S_8_). The ^1^H NMR spectra of the crude reaction mixtures show several resonances that are in agreement with the presence of multiple products. Among these products, we were able to crystallographically characterize a dimeric U(iv) persulfide complex, [(S_2_)U(OSi(O^*t*^Bu)_3_)_4_K_2_]_2_, and a dimeric tris(siloxide) U(iv) complex, {[UK(OSi(O^*t*^Bu)_3_)_3_]_2_(μ-S_2_)(μ-S_3_)}, containing both disulfide and trisulfide ligands (see ESI†). These results show that it is impossible to control the reaction stoichiometry by using elemental sulfur; thus, Ph_3_PS was used as the sulphur transfer agent.

**Scheme 1:**
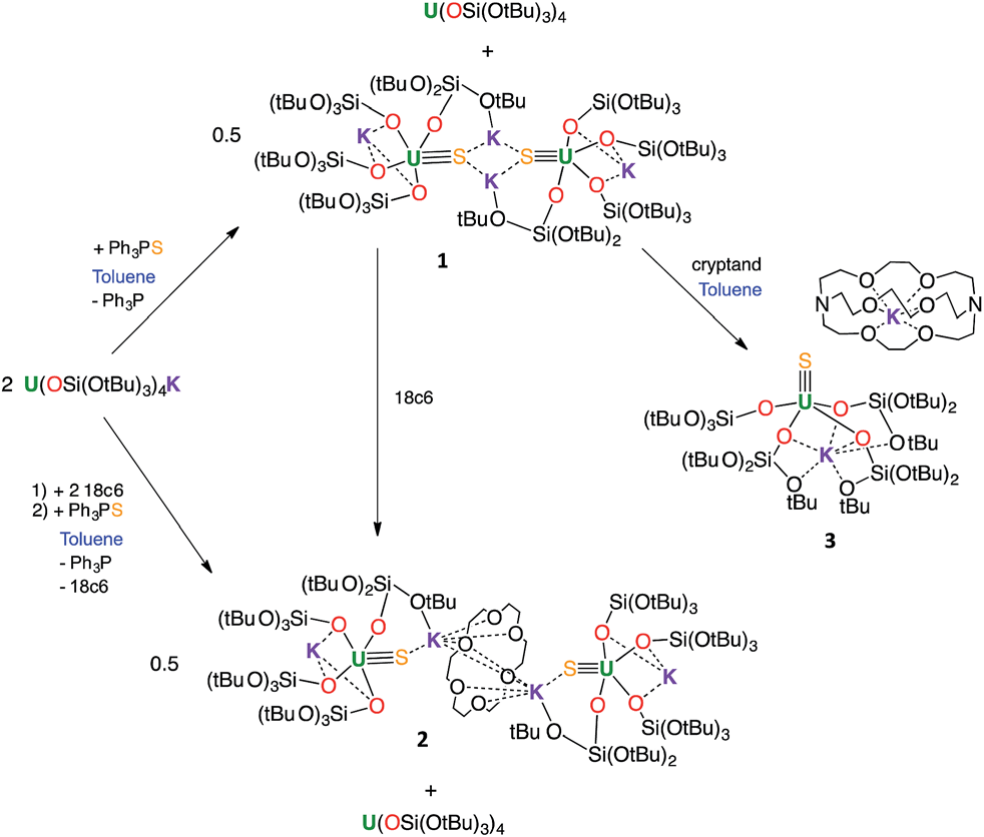
Synthesis of U(iv) terminal and potassium-capped sulfide complexes.

The reaction of 0.5 equivalents of Ph_3_PS with a brown solution of [U(OSi(O^*t*^Bu)_3_)_4_K] in toluene afforded a green solution from which the uranium(iv) sulfide complex [SU(OSi(O^*t*^Bu)_3_)_4_K_2_]_2_, **1** (Scheme 1), was isolated analytically pure with yields up to 62%. Proton NMR studies reveal that complex **1** is formed with similar conversion rates when [U(OSi(O^*t*^Bu)_3_)_4_K] is reacted with 1 or 0.5 equivalents of Ph_3_PS. The formation of **1** is accompanied by the formation of the uranium(iv) tetrasiloxide complex [U(OSi(O^*t*^Bu)_3_)_4_],^37^ which was identified by NMR spectroscopy. The reaction of the U(iii) complex [U(OSi(O^*t*^Bu)_3_)_4_K] with the two-electron oxidizing agent Ph_3_PS led to two different U(iv) products rather than one U(v) species. This is due to the fact that U(iii) complexes favour one-electron redox reactions to attain the thermodynamically more stable U(iv) ion. In this reaction, each Ph_3_PS oxidizes two U(iii) complexes to U(iv) and transfers the sulfur atom to one of the two tetrasiloxide complexes, generating the U(iv) sulfide complex **1**. Green single crystals of **1**·toluene were obtained from the crude reaction mixture at room temperature.

**Fig. 2:**
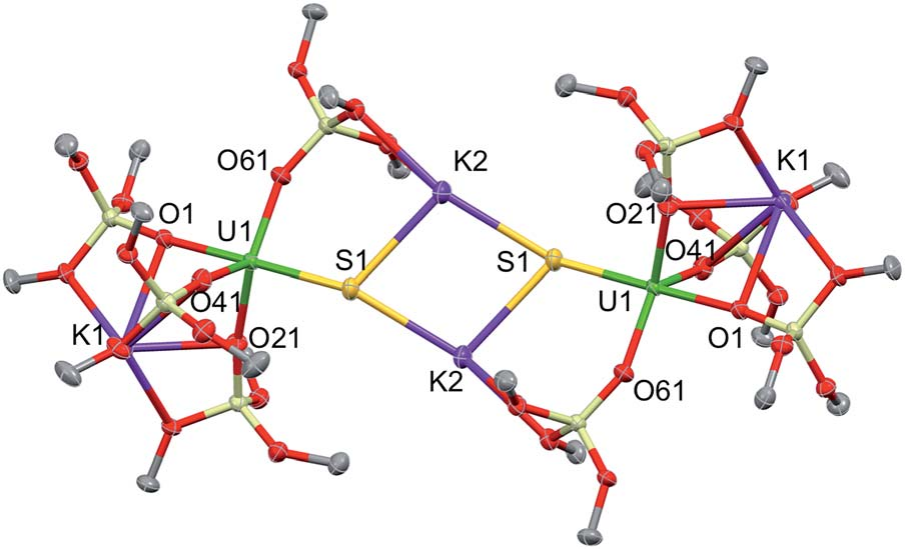
Thermal ellipsoid diagram of [SU(OSi(O^*t*^Bu)_3_)_4_K_2_]_2_ (**1**) (50% probability ellipsoids). The methyl groups and lattice solvent molecules are omitted for clarity.

Complex **1**·toluene crystallized as a dimer in the centrosymmetric, triclinic space group, *P*1[combining macron]. The solid-state structure of complex **1** (Fig. 2) shows that two [SU(OSi(O^*t*^Bu)_3_)_4_K_2_] moieties are bridged by two potassium cations, capping the sulfides to yield a dimer. Two potassium cations (each one also bound to a O^*t*^Bu group) and the two sulfides form an SKSK diamond core around the inversion centre. The second potassium ion of the asymmetric unit is located in an O6 coordination pocket formed by three siloxide ligands. The five-coordinate uranium centre is ligated by four oxygen atoms of the siloxide ligands and one sulfide. The coordination geometry can best be described as distorted trigonal bipyramidal. The U–S bond length (2.5440(8) Å) is significantly longer than those found in the previously reported sodium- or potassium-capped U(iv) sulfide complexes (2.4805(5)–2.4463(6) Å),^35^,^36^ probably due to steric hindrance. The S1–K2 bond length of 3.0455(12) Å is comparable to those found in the U(iv) complex [K(18c6)][U(S)(NR_2_)_3_] (R = SiMe_3_) (3.0684(8) Å and 3.1551(8) Å),^35^ in which the sulfide is capped by the K(18c6)^+^ cation. The average U–O_siloxide_ bond length (2.22(1) Å) falls in the range of U–O bond lengths reported for uranium(iv) siloxide complexes.^14^,^37^–^40^

The ^1^H NMR spectrum of crystals of **1** in deuterated toluene shows a very broad signal between 3 and 1 ppm, in agreement with the presence of a fluxional behaviour of the siloxide ligands in solution. Decreasing the temperature down to 233 K did not lead to a fully resolved spectrum. Complex **1** is stable in the solid state at room temperature but slowly decomposes in toluene at room temperature, leading to the formation of [U(OSi(O^*t*^Bu)_3_)_4_], free ligand and unidentified species (decomposition products are observed after 24 hours). Complex **1** can be isolated analytically pure from toluene due to its lower solubility compared to [U(OSi(O^*t*^Bu)_3_)_4_].

In order to identify the role of the potassium cation in the stabilization of the U(iv)–S species, and to prepare a terminal sulfide, complex 1 was reacted with 18c6 and 2.2.2-cryptand. The addition of 1 equivalent of 18c6 to **1** in toluene led to the formation of [{SU(OSi(O^*t*^Bu)_3_)_4_K_2_}_2_(μ-18c6)], **2**, containing a U(iv)–S group capped by a K(18c6)^+^ ion.

**Fig. 3:**
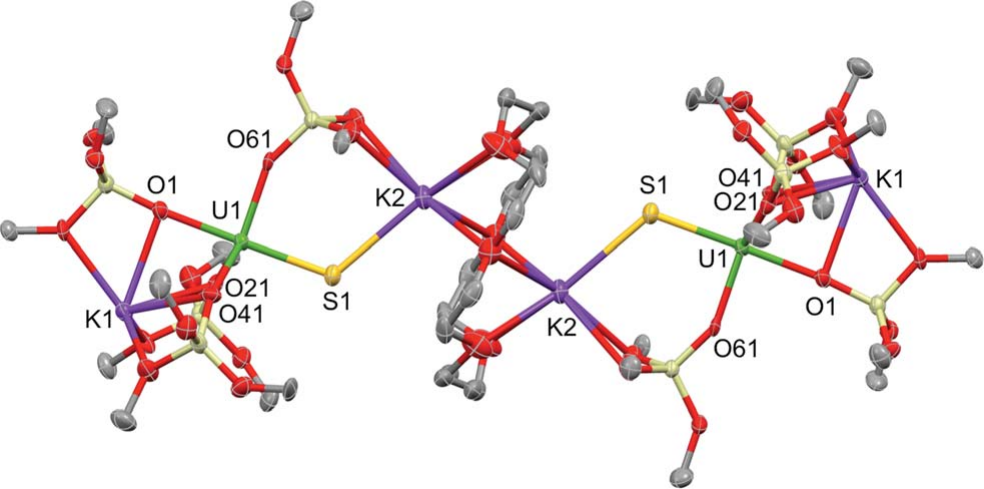
Thermal ellipsoid diagram of [{SU(OSi(O^*t*^Bu)_3_)_4_K_2_}_2_(μ-18c6)] (**2**·tol) (50% probability ellipsoids). Methyl groups and lattice solvent molecule are omitted for clarity.

Blue-green single crystals of **2** were obtained from a toluene solution of the reaction mixture at 233 K. **2**·tol crystallized in the centrosymmetric, monoclinic space group, *P*2_1_/*n*. In the structure of **2**, a 18c6 bridges two [SU(OSi(O^*t*^Bu)_3_)_4_K_2_] units to yield a dimer with the inversion centre located in the middle of the crown ether (Fig. 3). The coordination environment of the U(iv) ion in **2** is very similar to that found in **1**. In contrast, the US_2_K_2_ core present in the structure of **1** is disrupted by the presence of the bridging 18c6. Each potassium cation capping the sulfides in **2** is also bound to two O^*t*^Bu groups from a siloxide ligand and to four oxygen atoms of the bridging crown ether. Thus, the crown ether is coordinated to two different potassium cations, and adopts a non-planar conformation.

The U–S distance (2.534(2) Å) in **2** is similar to that found in complex **1**, indicating that the presence of the crown ether coordinated to the potassium cation does not significantly affect the U–S bonding interaction. The S1–K2 bond length (3.128(3) Å) in **2** is slightly elongated compared to **1** because of the presence of the crown ether (Table 1).

**Table 1:** Selected bond distances for complexes **1**, **2**, **3** and **5** (in Å)

Structural parameters	**1**·tol	**2**·tol	**3**·hex	**5**·tol
U–S	2.5440(8)	2.534(2)	2.5220(14)	2.834(3)
S–K	3.0455(12)	3.128(3)	—	3.229(5)
Av. U–O_bridging_	2.22(1)	2.22(2)	2.26(2)	—
Av. U–O_terminal_	—	—	2.197(4)	2.15(3)

Complex **2** can also be prepared by the reaction of 0.5 or 1 equivalent of Ph_3_PS with the U(iii) complex [U(OSi(O^*t*^Bu)_3_)_4_][K18c6] (Fig. 1, right)^37^ in toluene for 12 hours.

The ^1^H NMR spectrum of **2** in deuterated toluene features two signals for the siloxide ligands at –0.9 ppm and –10.3 ppm, respectively, with an integration ratio of 1 : 3. This is in agreement with the presence of a *C*_3_-symmetric species and a fluxional binding of the potassium cation in solution.

The solid-state structure of **2** shows that the addition of crown ether to complex **1** does not prevent the binding of potassium to the U(iv)–S. The addition of excess crown ether does not afford a more symmetric solution species, indicating that the binding of the potassium cannot be prevented by crown ether in solution.

In order to inhibit the coordination of potassium to the sulfide, we resorted to the use of 2.2.2-cryptand.

The addition of 1 equivalent of cryptand to a solution of **1** in toluene (Scheme 1) afforded the U(iv) terminal sulfide complex [SU(OSi(O^*t*^Bu)_3_)_4_K][Kcryptand], **3**.

**Fig. 4:**
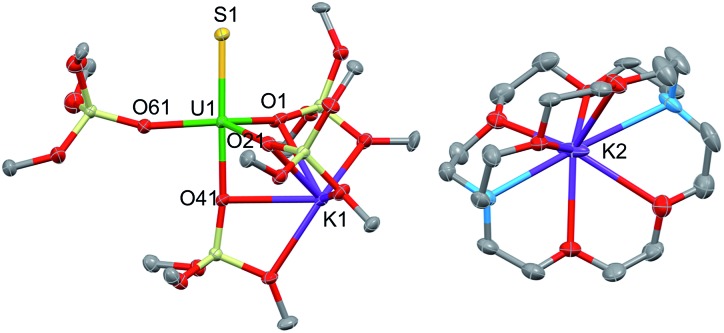
Thermal ellipsoid diagram of [SU(OSi(O^*t*^Bu)_3_)_4_K][Kcryptand] (**3**·hex) (50% probability ellipsoids). Methyl groups and lattice solvent molecule are omitted for clarity.

Green single crystals of **3** were obtained from hexane at room temperature. Complex **3**·hex crystallized in the centrosymmetric, triclinic space group, *P*1[combining macron], as a separated ion pair, consisting of the [Kcryptand]^+^ cation and the [SU(OSi(O^*t*^Bu)_3_)_4_K]^–^ anion (Fig. 4). In **3**, the five-coordinate uranium ion is ligated by four siloxide ligands and a terminal sulfide, giving a distorted trigonal bipyramidal coordination geometry. One potassium atom remains encapsulated in the O6 pocket made by three bridging siloxide ligands. The U–S bond length in **3** (2.5220(14) Å) is comparable to the U–S bond distance in complex **1** (2.5440(8) Å) (Table 1). This indicates that potassium binding and dimer formation only lead to a slight lengthening of the U–S bond. The U–O bond length is 2.197(4) Å for the terminal siloxide oxygen, while the average for the three UK-bridging siloxide ligands is 2.26(2) Å, which is in the range of previously reported U–O_siloxide_ bond lengths.^14^,^37^–^40^

The ^1^H NMR spectrum of **3** in deuterated toluene shows two signals (ratio 1 : 3) for the siloxide ligands, in agreement with a *C*_3_-symmetric species in solution within the NMR timescale. This suggests the presence of a unique chemical environment for the three siloxide ligands that compose the equatorial plane giving rise to one signal and one environment for the siloxide *trans* to the U=S giving rise to the second signal. This can be interpreted in terms of a fluxional binding of the potassium cation. Three additional paramagnetically shifted signals assigned to the cryptand protons are also observed. The paramagnetic shift of the cryptand signals strongly suggests that the terminal sulfide could be in fast exchange in solution with a potassium-capped sulfide species.^41^

The terminal sulfide complex **3** is stable in the solid state at room temperature, but slowly decomposes in toluene (50% decomposition after 1 month), as well as in THF solution (decomposition products already visible in the NMR spectrum after 24 hours), affording the U(iv) complex [U(OSi(O^*t*^Bu)_3_)_4_] and a mixture of other decomposition products. One of the decomposition products of **3** in THF has been identified as the U(iv)–U(iv) complex [S_3_U_2_(OSi(O^*t*^Bu)_3_)_6_K_3_][Kcryptand], **4** (20% conversion in **4** after 7 months determined by NMR spectroscopy). Complex **4** crystallizes as an ion pair consisting of the [U_2_(μ-S)_3_(OSi(O^*t*^Bu)_3_)_6_K_3_]^–^ anion and the [Kcryptand]^+^ cation. In the [U_2_(μ-S)_3_(OSi(O^*t*^Bu)_3_)_6_K_3_]^–^ anion, three S^2–^ anions bridge the two uranium atoms (see ESI†). Thus, the decomposition of complex **3** leads to the loss of one siloxide ligand from each uranium complex, as well as sulfide redistribution to afford a sulfide-bridged diuranium(iv) complex. Although a terminal U(iv) sulfide is stabilized by the presence of the sterically hindered environment provided by the four siloxide ligands, this complex can slowly eliminate one siloxide ligand and further react to afford a sulfide-bridged diuranium(iv) complex.

### Influence of cation binding on the S-transfer reaction

**Scheme 2:**
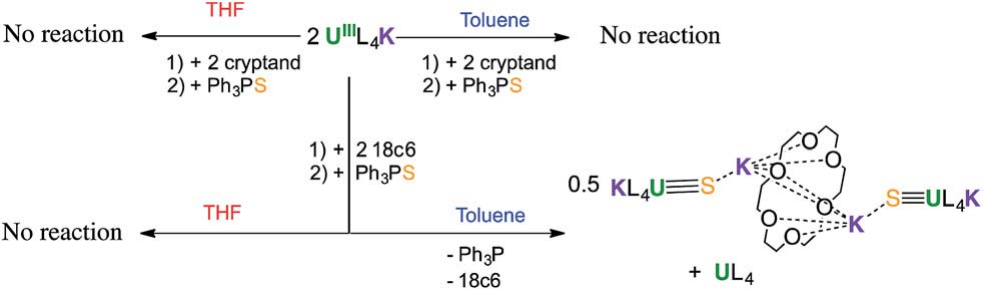
Reactivity of [U(OSi(O^*t*^Bu)_3_)_4_K] with Ph_3_PS in the presence of cryptand and 18C6.

Interestingly, no reaction is observed when Ph_3_PS is added to a solution of the U(iii) complex [U(OSi(O^*t*^Bu)_3_)_4_K] that has been pre-treated with cryptand (Scheme 2). This unambiguously shows that the presence of a bound potassium cation is crucial in the S-transfer reaction between [U(OSi(O^*t*^Bu)_3_)_4_K] and Ph_3_PS.

Notably, in toluene solution, the potassium cation remains bound to the siloxide ligands in complex [U(OSi(O^*t*^Bu)_3_)_4_K], while [U(OSi(O^*t*^Bu)_3_)_4_][Kcryptand] exists as separated ion pair. The solid-state structure of [U(OSi(O^*t*^Bu)_3_)_4_][K18c6]^37^ reveals an ion pair with (K18c6)^+^ located in the outer coordination sphere of the complex, but the coordination of potassium is probably still possible in solution.

To confirm the important role of potassium for the reactivity of the complexes, the reactions were studied in the more polar solvent THF. In THF, both [U(OSi(O^*t*^Bu)_3_)_4_][K18c6] and [U(OSi(O^*t*^Bu)_3_)_4_][Kcryptand] most likely exist as ion pairs and therefore do not react with Ph_3_PS. Reactivity is only observed in THF for the [U(OSi(O^*t*^Bu)_3_)_4_K] complex.

To some extent, such pronounced differences in reactivity arise from steric differences that result in reduced access of the substrate to the metal centre in [U(OSi(O^*t*^Bu)_3_)_4_][Kcryptand]. However, the possible role of cooperative binding of the potassium to the sulfur might also be important. Comparing the O–U–O angles in the reported X-ray structures of the heterobimetallic UK complex [U(OSi(O^*t*^Bu)_3_)_4_K] and the ion pair [U(OSi(O^*t*^Bu)_3_)_4_][K18c6],^37^ a significant difference is observed. In the ion pair [U(OSi(O^*t*^Bu)_3_)_4_][K18c6] the four ligands form a weakly distorted tetrahedron with three angles having a mean value of 110.6(6)° and three angles having a mean value of 108.3(3)°. In the UK complex, the coordination tetrahedron is highly distorted. The potassium cation coordinates three of the four ligands and brings them closer together, resulting in an average value of the three O–U–O angles of 94.91(7)°. In contrast, the O–U–O angles between the bridging siloxides and the terminal one are significantly larger (127.2(3)°, 122.5(3)° and 115.0(3)°) than those found in [U(OSi(O^*t*^Bu)_3_)_4_][K18c6], rendering the metal centre more accessible to the substrate.

In both complexes, the steric hindrance provided by the four siloxide ligands prevents the rapid formation of sulfide-bridged complexes previously observed when reacting neutral U(iii) silylamide- or tacn-based amido or aminophenolato complexes with sulfur transfer agents (S_8_ or Ph_3_PS).^17^–^19^,^21^,^42^

**Fig. 5:**
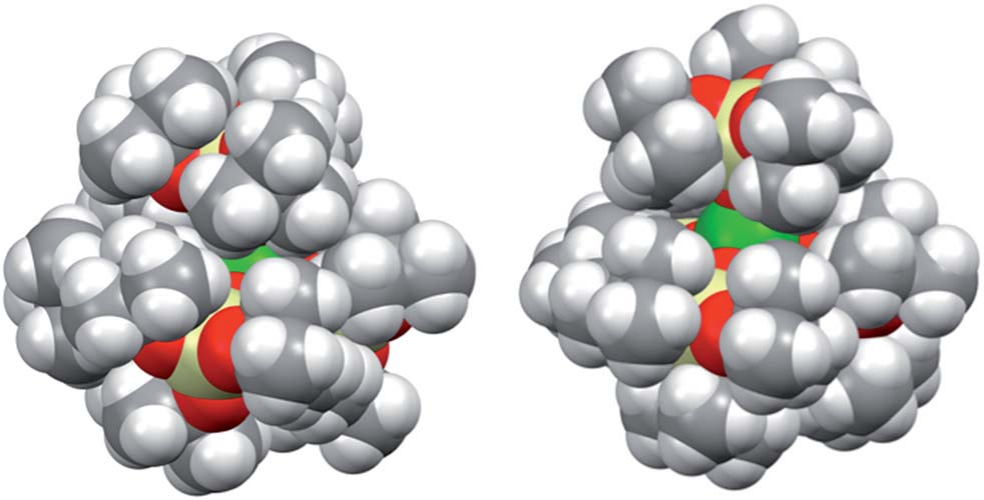
Space-filling representation of [U(OSi(O^*t*^Bu)_3_)_4_][K18c6] (left) and [U(OSi(O^*t*^Bu)_3_)_4_K] (right). The lattice solvent molecule and the [K18c6]^+^ cation in [U(OSi(O^*t*^Bu)_3_)_4_][K18c6] are omitted for clarity.

However, the presence of the potassium cation in the oxygen pocket of three siloxide ligands in the heterobimetallic UK complex results in easier access to the uranium centre than in the ion pair complex, in which the potassium is encapsulated in the crown ether (Fig. 1 and 5).

On the other hand, due to the low accessibility of the metal centre in these tetrasiloxide complexes, cooperative binding of potassium might be also important for the S-transfer process to occur. Binding of the sulfur atom to the potassium cation during the sulfur transfer reaction may also reduce the nucleophilic character of the sulfur, rendering reaction pathways leading to bridging sulfides less favourable. The important role of cooperative UK binding in the reduction of CO_2_ and CS_2_ by [U(OSi(O^*t*^Bu)_3_)_4_K] has been reported previously.^14^,^39^

### Reactivity of the U(iv) sulfide

**Scheme 3:**
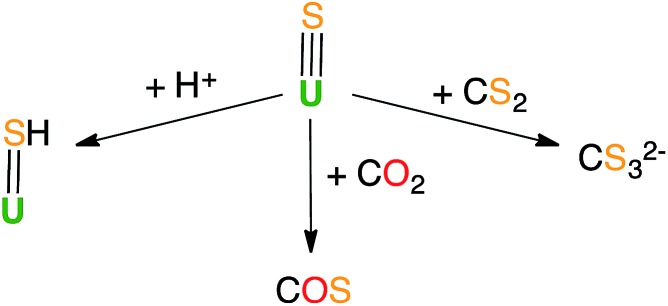
Reactivity of U(iv) sulfide species.

Previous reactivity studies have shown that sulfide-bridged diuranium(iv) complexes can undergo nucleophilic addition of chalcogens, CS_2_ and CO_2_, to afford stable disulfide-,^43^ trithiocarbonate-25 and monothiocarbonate-bridged (CO_2_S^2–^)^24^ diuranium(iv) complexes, respectively. In order to probe the possibility of sulfur transfer in complexes containing a U–S multiple bond, we investigated the reactivity of the sulfide complex **1** with various substrates (Scheme 3). To date, only chalcogen atom transfer reactivity has been investigated for terminal U(iv) sulfides, affording di-chalcogenide and tri-chalcogenide complexes depending on the stoichiometry.^44^

Complex **1** reacts rapidly with ^13^CS_2_ (1 equivalent per U atom) in toluene. The ^1^H NMR spectrum of the reaction mixture shows the presence of signals assigned to exchanging species that we were unable to identify due to the low stability of the corresponding compound.

After several hours, the presence of [U(OSi(O^*t*^Bu)_3_)_4_] was observed by ^1^H NMR spectroscopy. Dissolution of the evaporated reaction mixture in DMSO-*d*_6_ led to complete dissociation of the reaction products. The ^1^H NMR spectrum in DMSO-*d*_6_ only contains one signal, which was assigned to [U(OSi(O^*t*^Bu)_3_)_4_].

**Scheme 4:**
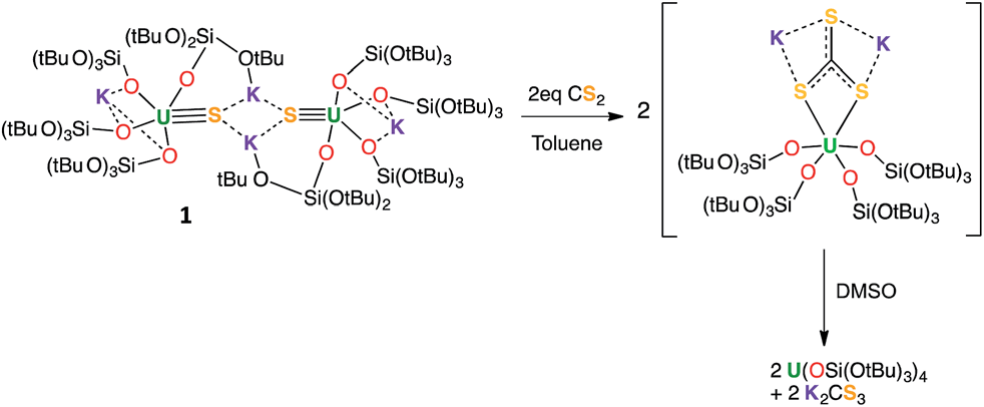
CS_2_ insertion into complex **1**.

The ^13^C NMR spectrum of the reaction products in DMSO-*d*_6_ shows the presence of only one signal at 267 ppm, which was assigned to free thiocarbonate.^45^ This indicates that, in toluene, the ^13^CS_2_ molecule inserts into the U–S bond to afford a U(iv) thiocarbonate complex (Scheme 4), which dissociates into [U(OSi(O^*t*^Bu)_3_)_4_] and K_2_CS_3_ in DMSO. The labile trithiocarbonate complex [U(OSi(O^*t*^Bu)_3_)_4_(μ_3_κ^2^:κ^2^:κ^2^CS_3_)K_2_(18c6)_2_]^39^ was isolated previously from the reaction of [U(OSi(O^*t*^Bu)_3_)_4_][K18c6] with CS_2_. This complex quickly dissociates in solution, affording [U(OSi(O^*t*^Bu)_3_)_4_] and K_2_CS_3_.

Complex **3** displays the same reactivity towards ^13^CO_2_ and ^13^CS_2_ as **1**. The ^13^C NMR spectrum in DMSO-*d*_6_ of the reaction of **3** with ^13^CS_2_ only shows the signals of thiocarbonate and 2.2.2-cryptand. The presence of the cryptand in the reaction mixture does not affect the insertion of CS_2_ into the U–S bond and a terminal thiocarbonate U(iv) complex is also likely to be formed in this case. Formation of a stable trithiocarbonate-bridged di-uranium(iv) complex from the nucleophilic addition of a sulfide-bridged diuranium(iv) complex to CS_2_ has been previously reported by Meyer *et al.*,^25^ but such reactivity has never been reported for terminal sulfides.

In light of the observed fast addition of CS_2_ to **1** and **3**, we also decided to explore the reaction of **1** with CO_2_. Complex **1** reacted immediately with an excess of ^13^CO_2_ in toluene, affording a new labile U(iv) species that decomposes rapidly at room temperature. Attempts to crystallize the reaction products were not successful. The ^13^C NMR spectrum of the reaction mixture in deuterated toluene shows the presence of a peak at 153.5 ppm that increases over time. This chemical shift corresponds to free COS.^46^ The formation of COS can be interpreted as arising from the decomposition of a hypothetical U–CO_2_S intermediate.

A sulfide complex seemed the ideal precursor for the straightforward synthesis of a U(iv) hydrosulfide complex and therefore we investigated the reactivity of complex **2** with pyHCl. This is a known strategy in transition metal chemistry for the synthesis of hydrosulfide complexes.^47^,^48^ Hydrosulfide complexes of transition metals have attracted considerable attention because of their relevance to metalloenzymes and metal sulfide catalysts for industrial hydrodesulfurization.^48^–^51^ The only crystallographically characterized uranium hydrosulfido complexes to date have been prepared through reduction of H_2_S by tacn- and N-anchored tris(aryl oxide) U(iii) complexes.^52^

**Fig. 6:**
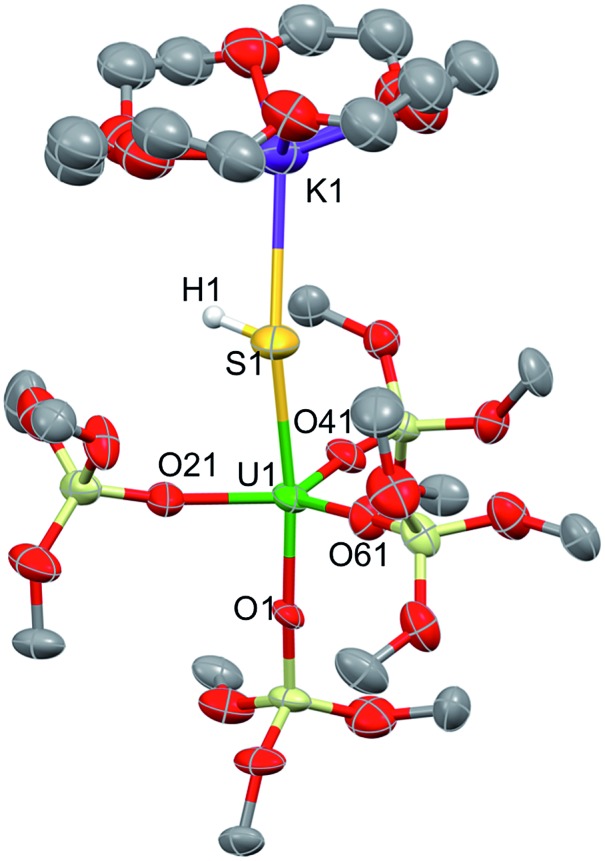
Thermal ellipsoid diagram of {[(SH)U(OSi(O^*t*^Bu)_3_)_4_][K18c6]} (**5**·tol) (50% probability ellipsoids). Methyl groups and lattice solvent molecule are omitted for clarity.

The addition of one equivalent (per U atom) of pyHCl to the [{SU(OSi(O^*t*^Bu)_3_)_4_K_2_}_2_(μ-18c6)] complex **2** in THF led to the formation of {[(SH)U(OSi(O^*t*^Bu)_3_)_4_][K18c6]}, **5**, with 53% conversion determined by ^1^H NMR spectroscopy. Light blue-green single crystals were obtained from the crude toluene mixture at 233 K (Fig. 6). Complex **5**·tol crystallized in the non-centrosymmetric, monoclinic space group, *Cc*. The five-coordinate uranium atom is ligated by four terminal siloxide ligands and one SH^–^ moiety in a distorted trigonal bipyramidal geometry. The 18c6-encapsulated potassium counter-ion is bound to the sulfur atom with a S1–K1 bond length of 3.229(5) Å. The U1–S1 distance was measured to be 2.834(3) Å, which is much longer than the U–S bond distance in **3** (2.5220(14) Å), but is very similar to that found in the only other example of a mononuclear U(iv)–SH complex that was reported by Meyer *et al.* (2.797(1) Å).^52^ The average U–O_siloxide_ bond length (2.15(3) Å) is similar in length to that found for the terminal siloxides in the terminal sulfide complex **3** (2.197(4) Å).

Complex **5** can also be prepared from the reaction of the U(iii) complex [U(OSi(O^*t*^Bu)_3_)_4_][K18c6] with H_2_S, but in much lower yield, independent of the U : H_2_S ratio (17% yield and 22% overall conversion was determined by quantitative NMR spectroscopy). Moreover, this alternative synthetic route requires the handling of a solution of toxic H_2_S. X-ray quality crystals of a dimeric by-product were also isolated from this reaction. This structure clearly shows the presence of two U(iv) and [U(OSi(O^*t*^Bu)_3_)_3_K] moieties, bridged by three sulfur atoms (complex **6** in ESI†). The bond valence sum and the intermediate average value of the U–S distances (2.83(2) Å) fall in between the reported values for bridging S^2–^ (2.59(1)–2.736(2) Å)^17^,^21^ and bridging SH^–^ (2.877(1)–2.964(1) Å)^52^ moieties, suggesting that 2 SH^–^ and 1 S_2_^2–^ groups are bridging the uranium centres. The hydrogens of the SH^–^ moieties are probably fluxional, and therefore very difficult to locate in the crystal structure. The isolation of this by-product suggests that the low yield of the reaction with H_2_S is probably associated with the fast reaction of the hydrosulfide with more than one equivalent of H_2_S (even using stoichiometric conditions). The further aggregation of the initially formed mononuclear hydrosulfide complexes leads to polynuclear sulfide complexes. This represents a well-recognized problem in the preparation of mononuclear hydrosulfide complexes.^48^ Therefore, the protonation of the sulfide **2** provides a more convenient route to the preparation of the hydrosulfide complex **5**.

### Computational bonding analysis

In order to investigate the nature of the U–S bond in complexes **1**, **3** and **6**, we performed calculations at the B3PW91 level. In particular for complex **1**, a small core pseudopotential basis set was chosen for the uranium atom, in which the f-electrons are included in the valence shell. Moreover, the monomeric form of complex **1** was considered for the sake of computational time. To verify the validity of such a theoretical protocol, we compared some important geometrical features to the available X-ray data and found that they are in close agreement (see ESI†). In particular, we found that the calculated values of the U–S bond distances were in agreement with the experimental ones (Fig. S.D.1†). Henceforth, we proceed into the analysis by firstly inspecting the related molecular orbitals (MOs).

**Fig. 7:**
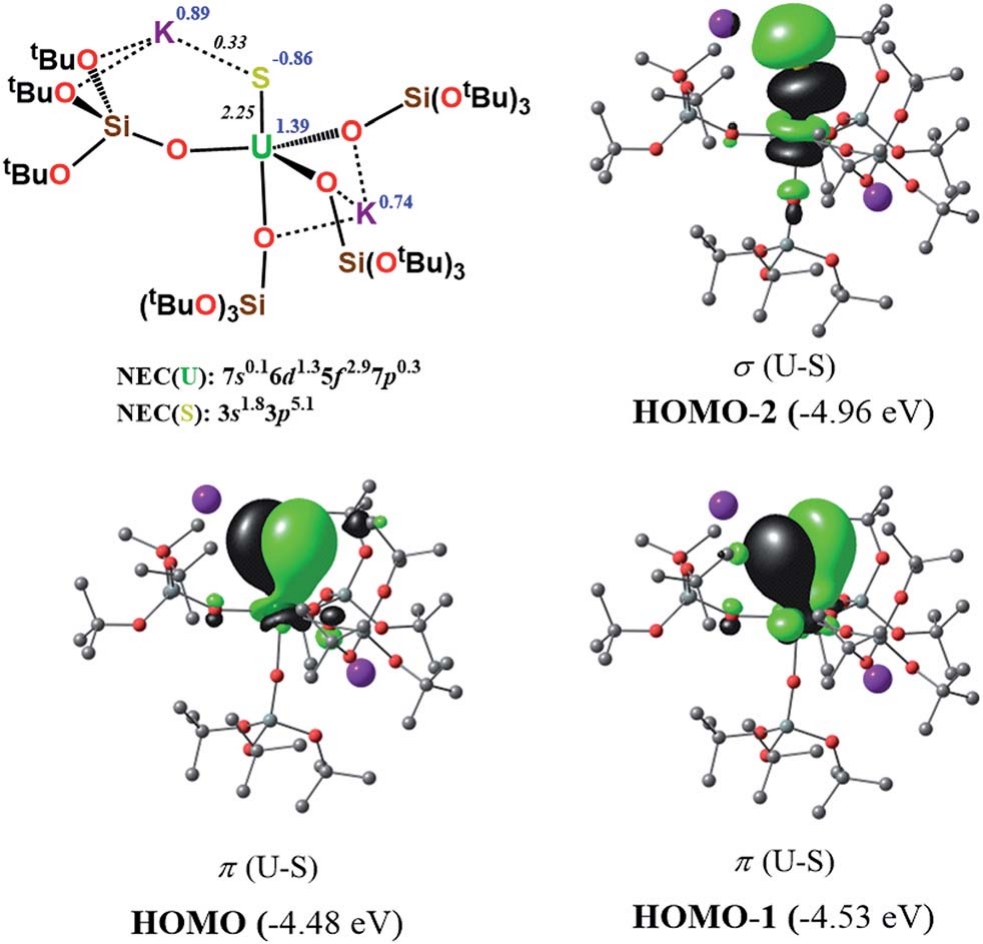
σ and π molecular orbitals (α-spin) calculated for the monomer-**1** using the small-core pseudopotential. The numbers in blue correspond to the natural charges and those in italics to the Wiberg bond orders. Hydrogen atoms are omitted for clarity.

As expected, for the U(iv) electron configuration (triplet multiplicity), the (SOMO)^α^ and (SOMO–1)^α^ orbitals correspond to pure non-bonding f-orbitals. Interestingly, the subsequent three MOs correspond to two π- (HOMO, HOMO–1) and one σ-type (HOMO–2) singly-occupied MOs of the U–S bond, as depicted in Fig. 9. It is worth noting that in (HOMO–2) the orbital is polarized towards the potassium atom, indicating small, but not negligible, overlap between the K and the S atom. This molecular orbital picture between the dianionic sulfide and the U(iv) centre, which is unconventional for the transition metals, is also found in H_2_U=S gas phase compounds.^33^ Similarly, they closely resemble the shape of the MOs responsible for the U≡N_terminal_ triple bond in Tren^TIPS^-based complexes,^53^,^54^ as well as of the recently reported Th(iv) chalcogenide tris(amide) system.^11^ The peculiar triple character bonding situation is also evident based on the natural bond orbital (NBO) analysis. In particular, the contribution of the uranium atom to the σ-bond is 18%, whereas the contribution of the sulfur is 77%. For the two π combinations, the contributions are 23% and 15% for the uranium atom, and 72% and 78% for the sulfur atom, respectively. Additionally, the two highest singly-occupied orbitals based on NBO analysis are almost pure 5f orbitals, with the composition of (SOMO–1)^α^ being 92% of U, and of (SOMO)^α^ being 84% of U and 8% of S. In the same way, the Wiberg bond order analysis in a Löwdin orthogonalized basis gave a bond order of 2.25 for the U–S bond, indicating partial triple bond character. In addition, the natural charges of the U and S are 1.39 and –0.86 |e|, respectively. The natural electron configuration (NEC) of the uranium (see Fig. 7) corresponds to what is expected for a formal f^2^ configuration. At first sight, this seems to indicate a crucial role of the f-orbitals in the bonding.

**Fig. 8:**
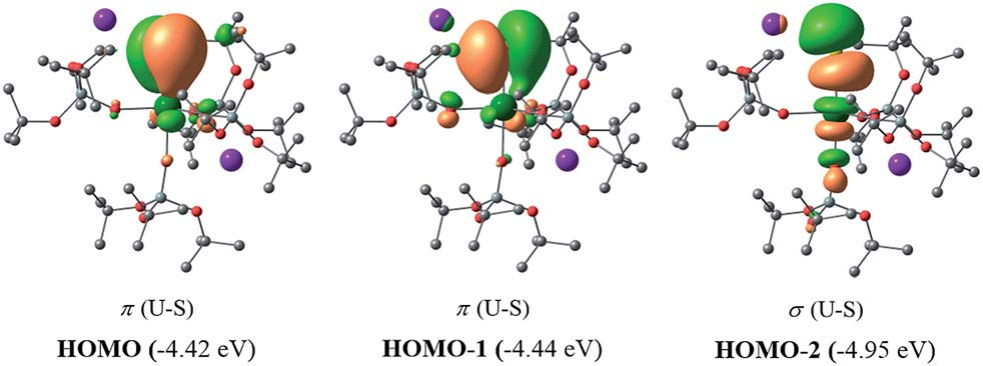
σ and π molecular orbitals (α-spin) calculated for the monomer-**1** using the “large-core” pseudopotential. Hydrogen atoms are omitted for clarity.

In order to further investigate the role of the f-orbitals in the bonding, we considered a different pseudopotential for the uranium(iv) atom, the so-called “large-core”. By using this f-in-core pseudopotential, the f-electrons are now explicitly included in the core shell configuration, and hence not available for any mixture with other orbitals, with the d-orbitals being the only ones available for bonding. However, even with such a computational strategy, the bonding picture remains essentially the same, as illustrated in Fig. 8. It should be noted that the NBO analysis also predicts that the U–S bond features triple-bond character as well, in line with the corresponding small-core calculations.

Therefore, the triple-bond character is not due to 5f involvement in the bonding. Finally, the spin density (SD) of uranium was found to be 2.19 (being depopulated by 0.19), with most of this residual being donated to the sulfide atom (SD^sulfur^ = –0.12).

**Fig. 9:**
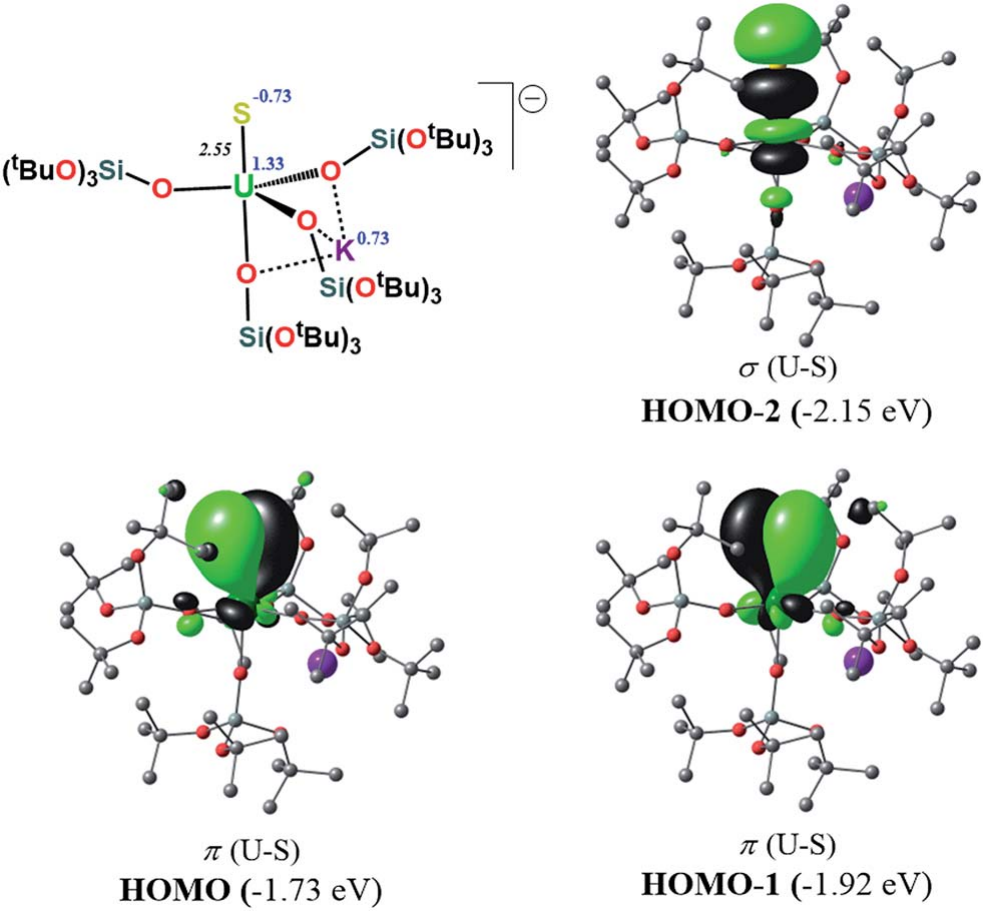
σ and π molecular orbitals (α-spin) calculated for **3**, using the small-core pseudopotential. The numbers in blue correspond to the natural charges and those in italics to the Wiberg bond orders. Hydrogen atoms are omitted for clarity.

In order to investigate the effect of the presence of S-bound potassium on the U–S bond, we also calculated the electronic structure of complex **3**. The cryptand moiety was excluded from the calculations, and consequently a negative charge was placed on the overall complex. The DFT-predicted structure of the triplet state, which is imposed by the uranium +IV oxidation state, is in close agreement with that found in the solid-state structure (see ESI†). Molecular orbital analysis gave the same picture for the bonding situation between the U and S atoms as for the monomeric structure **1**, as clearly depicted in Fig. 9.

Interestingly, despite the absence of a second potassium atom in the vicinity of the sulfide, the total picture of the bonding remains unchanged with respect to monomer-**1**. Minor differences are apparent, which are mostly linked to the absence of the polarization induced by the potassium atom on the σ-orbital (HOMO–2), and to the slightly smaller natural charge located on the sulfide group. Moreover, there are virtually no changes to the charges of the uranium and potassium atom in **3** with respect to the corresponding ones in monomer-**1**. NBO analysis also predicted a σ^2^π^4^ U–S configuration, in line with a triple bond. Specifically, the σ-bond has 20% uranium and 76% sulfur character, whereas the contributions of each atom to the two π bonds are 29/18% for U and 67/75% for S, respectively. Again, as found for monomer-**1**, the exclusion of the f-electrons from the valence shell does not change the bonding picture (see ESI†). The bond order of the U–S bond was found to be 2.55, slightly higher than that calculated for monomer-**1**. In addition, the spin density (SD) of uranium was found to be 2.21 and that of the sulfur was found to be –0.14.

**Fig. 10:**
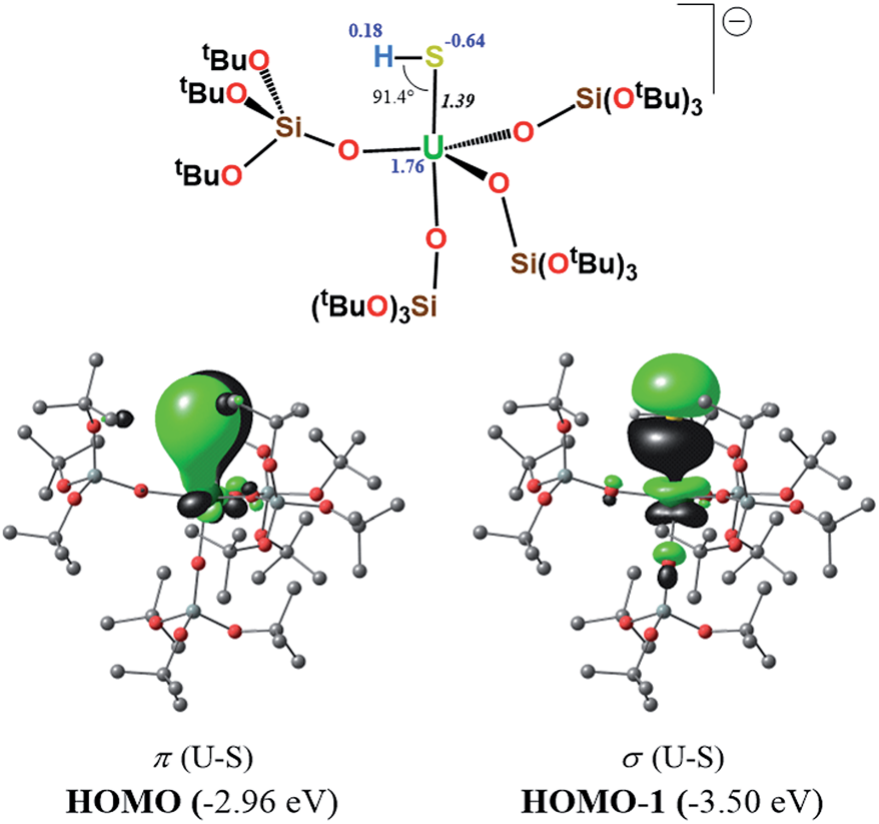
σ and π molecular orbitals (α-spin) calculated for complex **5** using the small-core pseudopotential. The numbers in blue correspond to the natural charges and those in italics to the Wiberg bond orders. Hydrogen atoms are omitted for clarity.

In order to gain insights into the bonding situation in complex **5**, we proceeded to the optimization of the X-ray structure at the B3PW91 level, once again using the “small-core” basis set for the uranium atom. The potassium crown ether (K18C6) cation was excluded from the calculations, and consequently a negative charge was placed on the overall complex. Molecular orbital analysis is consistent with the presence of a double bond between the U and S atoms, as depicted in Fig. 10. This is in line with the previous MO picture found in the monomer-**1** and **3** models, since here the protonation of the strongly nucleophilic sulfide results in the breaking of one of the two π bonds. In particular, the HOMO–1 and HOMO orbitals possess 14% and 12% uranium character, respectively, and 82% sulfur character in both cases. The two SOMOs are mainly composed of pure f-orbitals. This further highlights the strongly polarized nature of this bond. Interestingly, the Wiberg bond orders in a Löwdin orthogonalized basis gave a bond order of 1.39 for the U–S bond, a value that is significantly smaller than in the other complexes, and is fully consistent with the partial double character of such an interaction.

## Conclusions

In summary, we have isolated a new series of complexes containing a U–S bond from the reaction of the bulky U(iii) tetrasiloxide complex [U(OSi(O^*t*^Bu)_3_)_4_K] with Ph_3_PS.

This is the first example of a terminal uranium(iv) sulfide complex, which was directly obtained from the reaction of U(iii) and a sulfur-transfer agent without the addition of protecting groups. The bulk provided by the four siloxide ligands prevents the formation of sulfide-bridged complexes. Moreover, the presence of bound potassium is essential for the reaction to occur, probably due to both steric effects and cooperative binding of the sulfur-transfer reagent to the K and U centres.

The terminal sulfide can easily be transferred to CO_2_ and CS_2_ to afford new thiocarbonates. Moreover, the terminal sulfide provides a convenient precursor for the synthesis of the corresponding hydrosulfide complex upon protonation with PyHCl.

DFT calculations carried out for the potassium-bound sulfide complex **1**, and for the terminal sulfide complex [SU(OSi(O^*t*^Bu)_3_)_4_K][Kcryptand], **3**, showed that the U–S interactions in both complexes consist of three bonding pairs (σ + 2π bonds) with a Wiberg bond order of 2.25 for **1** and a bond order of 2.55 for **3**. However, the use of the “large-core” pseudopotential indicates that the triple-bond character is not due to 5f involvement in the bonding. For the hydrosulfide complex **5**, the molecular orbital analysis is consistent with the presence of a double bond between the U and S atoms with a Wiberg bond order of 1.39, a value significantly smaller than those found in complexes **1** and **3**.

Future studies will be directed to explore the reactivity of the terminal sulfide with other organic molecules and to isolate terminal sulfide complexes containing uranium in oxidation states higher than +IV and to investigate the nature of the U–S bond in these systems.

## Experimental

### General procedures

Unless otherwise noted, all experiments were carried out at ambient temperature under an inert atmosphere using Schlenk techniques and an MBraun glovebox equipped with a purifier unit. Water and oxygen levels were always kept at less than 1 ppm. Glassware was dried overnight at 150 °C prior to use. Syntheses were performed using glass-covered stirring bars.

### Starting materials

Solvents were purchased in their anhydrous form from Aldrich or Cortecnec (deuterated solvents), conditioned under argon and vacuum distilled from K/benzophenone (toluene, THF) or sodium dispersion (hexane) or dried over molecular sieves for one week (DMSO). All reagents were dried under high-vacuum for 5 days prior to use. Dry ^13^CO_2_ was prepared by storing over molecular sieves for one week prior to use. HOSi(O^*t*^Bu)_3_ ligand was purchased from Aldrich and purified by sublimation prior to use. Depleted uranium turnings were purchased from the “Société Industrielle du Combustible Nucléaire” of Annecy (France). [U(OSi(O^*t*^Bu)_3_)_4_K]^14^ and [U(OSi(O^*t*^Bu)_3_)_4_][18c6]^37^ were prepared according to the published procedures.

### ^1^H NMR experiments

NMR spectra were performed in J. Young NMR tubes. ^1^H and ^13^C NMR spectra were recorded on a Bruker 400 MHz spectrometer. NMR chemical shifts are reported in ppm and are referenced to the residual ^1^H and ^13^C signals of the deuterated solvents.

### Elemental analyses

They were performed under argon by Analytische Laboratorien GMBH at Lindlar (Germany) or by the elemental analyses department of the EPFL using a Thermo Scientific Flash 2000 Organic Elemental Analyzer.

### X-ray analyses

Experimental details for X-ray data collections of all complexes are given in Table S1.† Figure graphics were generated using MERCURY 3.6 supplied by the Cambridge Structural Database; CCDC: Cambridge, U.K., 2001–2015. Diffraction data were taken using Oxford-Diffraction Xcalibur S or Bruker APEX II CCD kappa geometry diffractometers (Mo-Kα radiation, graphite monochromator, *λ* = 0.71073 Å). To prevent evaporation of co-crystallized solvent molecules the crystals were coated with light hydrocarbon oil and the data were collected at 150 K or 100 K. The datasets were reduced by CrysAlis^55^ or EvalCCD^56^ and then corrected for absorption.^57^

The structure resolutions were performed with SHELXS or Superflip and the structure refinement was performed with SHELXL.^58^,^59^ The crystal structures were refined using full-matrix least-squares based on *F*^2^ with all non-hydrogen atoms anisotropically defined. Hydrogen atoms were placed in calculated positions by means of the “riding” model. Additional electron density found in the difference Fourier map (due to highly disordered solvent) was eventually treated by the SQUEEZE algorithm of PLATON.^60^

### Synthesis of complex **1**

A colourless solution of PPh_3_S (26.3 mg, 0.089 mmol, 0.5 eq.) in toluene (2 mL) was added to a stirred brown solution of [U(OSi(O^*t*^Bu)_3_)_4_K] (238.1 mg, 0.179 mmol, 1 eq.) in toluene (4 mL). The mixture was stirred at room temperature for 18 h. The resulting green solution was concentrated to approximately 3 mL and big green crystals formed overnight from toluene at room temperature. The crystals were filtered and dried for 2 hours (79.3 mg, 62% yield in 2 crops). The yield can be increased by recovering additional crops but it leads to co-crystallization of small amounts of the byproduct [U(OSi(O^*t*^Bu)_3_)_4_]. ^1^H NMR (400 MHz, Tol-*d*_8_, 298 K): *δ* [ppm] ≈ 1 (very broad, 216H). Anal. calcd for **1**·(tol)_0.6_ C_100.2_H_220.8_O_32_Si_8_S_2_K_4_U_2_: C, 42.09; H, 7.78; S, 2.24. Found C, 42.17; H, 7.68; S, 2.10. Green single crystals of **1**·tol suitable for X-ray diffraction were obtained from a concentrated toluene solution of the complex at room temperature.

### Reaction of [U(OSi(O^*t*^Bu)_3_)_4_][K18c6] with 0.5 eq. of Ph_3_PS: isolation of **2**

A colourless solution of PPh_3_S (2.1 mg, 0.007 mmol, 0.5 eq.) in toluene (1 mL) was added to a stirred brown suspension of [U(OSi(O^*t*^Bu)_3_)_4_][K18c6] (22.3 mg, 0.014 mmol, 1 eq.) in toluene (0.5 mL). The mixture was stirred at room temperature for 18 h to yield a green solution. The ^1^H NMR spectrum shows the presence of PPh_3_, [U(OSi(O^*t*^Bu)_3_)_4_] and complex **2** (90% conversion determined by ^1^H NMR spectroscopy using naphthalene as an internal standard) in the reaction mixture. ^1^H NMR (400 MHz, Tol-*d*_8_, 298 K): *δ* [ppm] = –0.9 (s, 162H), –10.3 (s, 54H). Blue-green single crystals of **2**·tol were obtained from the toluene reaction mixture at 233 K. Due to the similar solubility of [U(OSi(O^*t*^Bu)_3_)_4_] and complex **2**, **2** can only be isolated in low yield from this reaction (25%).

Complex **2** can also be obtained with similar conversion rates by addition of 1 eq. of 18c6 per U atom to complex **1** in toluene.

Anal. Calcd for **2** C_108_H_240_O_38_Si_8_S_2_K_4_U_2_: C, 42.28; H, 7.88; found C, 42.33; H, 8.07.

### Synthesis of complex **3**

A colourless solution of 2.2.2-cryptand (7.8 mg, 0.021 mmol, 2 eq., 1 eq. per U) in toluene (1 mL) was added to a stirred green solution of complex **1** (29.5 mg, 0.010 mmol, 1 eq.) in toluene (1 mL). After 20 min. of stirring, toluene was removed and hexane (1 mL) was added. Green single crystals of complex **3**·hex formed from hexane at room temperature. The crystals were filtered, washed with hexane and dried under vacuum for 1 h (27 mg, 74%). ^1^H NMR (400 MHz, Tol-*d*_8_, 298 K): *δ* [ppm] = 13.1 (bs, 12H), 12.9 (bs, 12H), 11.8 (bs, 12H), –0.5 (s, 81H), –10.6 (s, 27H). Anal. calcd for **3** C_66_H_144_N_2_O_22_Si_4_SK_2_U: C, 44.57; H, 8.16; N, 1.58. Found C, 44.16; H, 8.15; N, 1.65.

### Reaction of complex **1** with **2** eq. of ^13^CS_2_

To a green solution of complex **1** (8.5 mg, 0.003 mmol, 1 eq.) in deuterated toluene (0.5 mL), 9.3 μL of a 636.5 mM solution of ^13^CS_2_ (0.006 mmol, 2 eq.) in deuterated toluene was added. The reaction mixture immediately turned light yellow, affording a labile species. ^1^H NMR (400 MHz, Tol-*d*_8_, 298 K): *δ* [ppm] = 11.1 (bs, 27H), –2.8 (bs, 81H). The solution was periodically monitored by ^1^H NMR spectroscopy over the course of a week, and it showed a decrease in the intensity of the two broad peaks and an increase in the intensity of the signal corresponding to the U(iv) complex [U(OSi(O^*t*^Bu)_3_)_4_]. This evolution shows the lability of the likely formed thiocarbonate complex, resulting in the release of the thiocarbonate anion. When toluene was removed and DMSO-*d*_6_ added, the ^13^C NMR spectrum only showed the signal assigned to thiocarbonate. ^13^C{^1^H} NMR (100 MHz, DMSO-*d*_6_, 298 K): *δ* [ppm] = 267.4 (s, CS_3_^2–^).

### Reaction of complex **1** with an excess of ^13^CO_2_

An excess (1 atm) of ^13^CO_2_ was added to a frozen green solution of complex **1** (11.7 mg, 0.004 mmol, 1 eq.) in deuterated toluene (0.5 mL). The solution was allowed to warm up to room temperature to yield a light pink solution. ^1^H NMR (400 MHz, Tol-*d*_8_, 298 K): *δ* [ppm] = 8.7 (bs, 27H), –2.9 (bs, 81H). ^13^C{^1^H} NMR (100 MHz, Tol-*d*_8_, 298 K): *δ* [ppm] = 153.5 (s, CSO).

### Synthesis of complex **5**

A colourless solution of 18c6 (5.2 mg, 0.020 mmol, 2 eq.) in THF (1 mL) was added to a stirred green solution of complex **1** (28.2 mg, 0.010 mmol, 1 eq.) in THF (1 mL). The resulting green solution of complex **2** was stirred for ten minutes and was added to a stirred white suspension of PyHCl (2.3 mg, 0.020 mmol, 2 eq.) in THF (1 mL). The resulting yellow suspension was stirred for 2 h to yield a light green suspension. The ^1^H NMR spectrum of the crude reaction mixture in THF-*d*_8_ at 298 K showed the formation of complex **5** with 53% conversion (determined by NMR spectroscopy using naphthalene as an internal standard) as the main reaction product. Blue-green single crystals of complex **5**·tol were obtained by storing the toluene reaction mixture at 233 K. The crystals were collected and dried under vacuum for 2 h (11.1 mg, 34% yield). ^1^H NMR (400 MHz, THF-*d*_8_, 298 K): *δ* [ppm] = 3.07 (s, 24H, 18c6), 0.74 (s, 108H). Anal. calcd for **5**·(tol)_0.5_ C_63.5_H_137_O_22_Si_4_SKU: C, 45.55; H, 8.25. Found C, 45.66; H, 8.61.

Complex **5** was also obtained in lower yield (17%) (22% overall conversion determined by NMR spectroscopy using naphthalene as an internal standard) by addition of 1 eq. of H_2_S to the U(iii) complex [U(OSi(O^*t*^Bu)_3_)_4_][K18c6] in THF.

The reaction of complex **1** with PyHCl was carried out under analogous conditions. The ^1^H NMR spectrum of the crude reaction mixture performed in THF-*d*_8_ at 298 K after addition of 18c6 showed the formation of **5** with 48% conversion.

The reaction of **3** with PyHCl affords {[(SH)U(OSi(O^*t*^Bu)_3_)_4_][Kcrypt]} with 52% conversion as determined by ^1^H NMR. ^1^H NMR (400 MHz, THF-*d*_8_, 298 K): *δ* [ppm] = 3.36–3.33 (m, crypt), 2.35 (s, crypt), 0.70 (s, 108H) (the identity of this complex was confirmed by comparing this ^1^H NMR spectrum to that obtained after adding cryptand to a solution of complex **5**).

### Computational details

All the structures reported in this study were fully optimized with the Becke's 3-parameter hybrid functional combined with the non-local correlation functional provided by Perdew/Wang (denoted as B3PW91).^61^,^62^ The Stuttgart-Dresden RECP (relativistic effective core potential) 5f-in-valence was used for the uranium atom, in combination with its adapted basis set.^63^–^65^ However, in some cases, the 5f-in-core ECP augmented by a f polarization function (*α* = 1.0) was used for the fixed oxidation state IV of the uranium atom.^66^ In addition, silicon atoms were treated with the corresponding Stuttgart-Dresden RECP in combination with its adapted basis sets,^67^ each one augmented by an extra set of polarization functions.^68^ For the rest of the atoms, the 6-31G(d,p) basis set was used.^69^–^71^ For analysing the bonding situation in the complexes of interest, we mainly used natural bond orbital analysis (NBO) using Weinhold's methodology.^72^,^73^ Also, the Multiwfn program^74^ was used for obtaining the composition of the molecular orbitals, based on the natural atomic orbital method,^75^ as well as the Wiberg bond order analysis in a Löwdin orthogonalized basis. The Chemcraft program was used for the visualization of the molecular orbitals.^76^ Finally, the GAUSSIAN09 program suite was used in all calculations.^77^

## Supplementary Material

Supplementary information

Crystal structure data
