# Supplementary material for: Synthesis and reactivity of a terminal uranium(iv) sulfide supported by siloxide ligands
Source: Chem Sci. 2016 May 10;7(9):5846–56. doi: 10.1039/c6sc00675b (PMC6024242; doi:10.1039/c6sc00675b)
Supplement: Supplementary file 1 [file SC-007-C6SC00675B-s001.pdf]

Electronic Supplementary Material (ESI) for Chemical Science.
This journal is © The Royal Society of Chemistry 2016

Supporting Information for the Manuscript

Synthesis and reactivity of a terminal uranium(IV) sulfide supported by siloxide ligands

Julie Andrez,[a] Jacques Pécaut,[b] Rosario Scopelliti,[a] Christos E. Kefalidis,[c] Laurent Maron,[c] Michael W. Rosenzweig,[d] Karsten Meyer [d] and Marinella Mazzanti*[a]

[a] Institut des Sciences et IngénierieChimiques, Ecole Polytechnique Fédérale de Lausanne (EPFL), 1015 Lausanne, Switzerland.

E-mail: marinella.mazzanti@epfl.ch

[b] Univ. Grenoble Alpes, INAC-SCIB, F-38000 Grenoble, France.
CEA, INAC-SCIB, Reconnaissance Ionique et Chimie de Coordination, 17 rue des Martyrs, F-38054 F-38054 Grenoble Cedex 09.

[c] Université de Toulouse et CNRS INSA, UPS, CNRS, UMR 5215, LPCNO, 135 avenue de Rangueil, 31077 Toulouse, France.

[d] Department of Chemistry and Pharmacy, Inorganic Chemistry, Friedrich-Alexander University Erlangen-Nürnberg (FAU), Egerlandstr. 1, 91058 Erlangen, Germany.

*Correspondence to Dr. Marinella Mazzanti

Contents:

A) Syntheses

B) ^{1}H NMR spectra

C) X-ray crystallographic data

D) Computational results

A) Syntheses

Isolation of $[(S_2)U(OSi(OtBu)_3)_4K_2]_2$

A brown solution of $[U(OSi(OtBu)_3)_4K]$ (83.0 mg, 0.0624 mmol, 1 eq.) in deuterated toluene (0.5 mL) was added onto elemental sulphur (2.0 mg, 0.0078 mmol, 0.125 eq.), stirred 18 hours and then sonicated 10 minutes. The reaction mixture turned light brownish. The ^1H NMR spectrum of the crude mixture in Tol-d_8 shows the presence of free $KOSi(OtBu)_3$ (1.44 ppm), uranium(IV) complex $[U(OSi(OtBu)_3)_4]$ (1.07 ppm) and of two unknown species at -1.38 ppm and 0.44 ppm. Suitable crystals for X-ray diffraction of $[(S_2)U(OSi(OtBu)_3)_4K_2]_2$.tol were obtained from the toluene reaction mixture at 233 K.

Isolation of $\{[UK(OSi(OtBu)_3)_3]_2(\mu\text{-}S_2)(\mu\text{-}S_3)\}$

A brown solution of $[U(OSi(OtBu)_3)_4K]$ (76.7 mg, 0.0577 mmol, 1 eq.) in toluene (2 mL) was added onto elemental sulfur (3.7 mg, 0.0014 mmol, 0.25eq), and stirred for 18 hours. The solvent was removed under reduced pressure and the resulting solid was washed with hexane, centrifuged and dried under vacuum. The ^1H NMR spectrum of the obtained solid in Tol-d_8 shows the presence of a peak at 0.44 ppm as the main species. Suitable crystals for X-ray diffraction of $\{[UK(OSi(OtBu)_3)_3]_2(\mu\text{-}S_2)(\mu\text{-}S_3)\}$**.**tol were obtained from the crude reaction solution at 233 K. It is noteworthy, that the ^1H NMR shift of complex $\{[UK(OSi(OtBu)_3)_3]_2(\mu\text{-}S_2)(\mu\text{-}S_3)\}$ is similar to **5,** even if the disulfide and trisufide ratios are different in these two structures.

Decomposition of 1 in THF: isolation of complex 4.

A colorless solution of 2.2.2-cryptand (3.8 mg, 0.010 mmol, 2 eq., 1 eq. per U) in THF (0.3 mL) was added to a stirred green solution of complex **1** (14.4 mg, 0.005 mmol, 1 eq.) in THF (0.2 mL). After 10 min. of stirring, the green solution was layered with hexane and was allowed to stand for one week at room temperature. Then the mixture was cooled down to 233 K, resulting in the formation of brown single crystals overnight. ^1H NMR (400 MHz, THF-d_8, 298 K): $\delta = 1.36$ ppm (s, 162H). The formation of the U(IV) complex, $[U(OSi(OtBu)_3)_4]$, was also identified as decomposition product by ^1H NMR spectroscopy. The observed U/S ratio of 2:3 in **4** (compared to a U/S ratio of 1:1 in **3**) requires the presence of other decomposition products that remain unidentified.

Reaction of complex 1 with 0.25 eq. of S_8

A green solution of complex **1** (17.8 mg, 0.006 mmol, 1 eq.) in toluene (1 mL) was added to a stirred yellow suspension of S_8 (0.4 mg, 0.0015 mmol, 0.25 eq.) in toluene (1 mL), resulting in a brown solution. After 1.5 h of stirring, a ^1H NMR spectrum was recorded (400 MHz, Tol-d_8, 298 K) and it showed the formation of the complex $\{[UK(OSi(OtBu)_3)_3]_2(\mu\text{-}S_2)(\mu\text{-}S_3)\}$ (0.44 ppm) with 36% conversion (determined by NMR spectroscopy using naphthalene as an internal standard) among other reaction products. Brown single crystals of this complex were obtained from the toluene reaction mixture at 233 K. The same compound was also obtained together with other unidentified products from the reaction of **1** with 0.125 eq. of S_8 in toluene. Attempts to isolate $\{[UK(OSi(OtBu)_3)_3]_2(\mu\text{-}S_2)(\mu\text{-}S_3)\}$ analytically pure failed due to the presence of other reaction products.

X-ray diffraction on crystal of $\{[UK(OSi(OtBu)_3)_3]_2(\mu\text{-}S_2)(\mu\text{-}S_3)\}$ show that the unit cell contains two complexes and in both complexes two $[UK(OSi(OtBu)_3)_3]$ moieties are bridged by disordered S_3^{2-} and S_2^{2-} ligands (occupancy factor of 0.451(6) and 0.549(6), respectively). Accordingly, chalcogen addition is accompanied by loss of one siloxide ligand. However, a mixture of complexes (U-(μ□S_3)$_2$-U, U-(**μ**□S_2)$_2$-U and U-(**μ**□S_3)(□**μ**□S_2)-U) cannot be ruled out.

B) ^{1}H NMR spectra

Figure S.B.1. ^{1}H NMR spectra of the 1:0.5 reaction mixture of [U(OSi(OtBu)$_3$)$_4$] and Ph$_3$PS (top) and of [US(OSi(OtBu)$_3$)$_4$K$_2$]$_2$ in anhydrous toluene-d_8 (200 MHz, 298K).

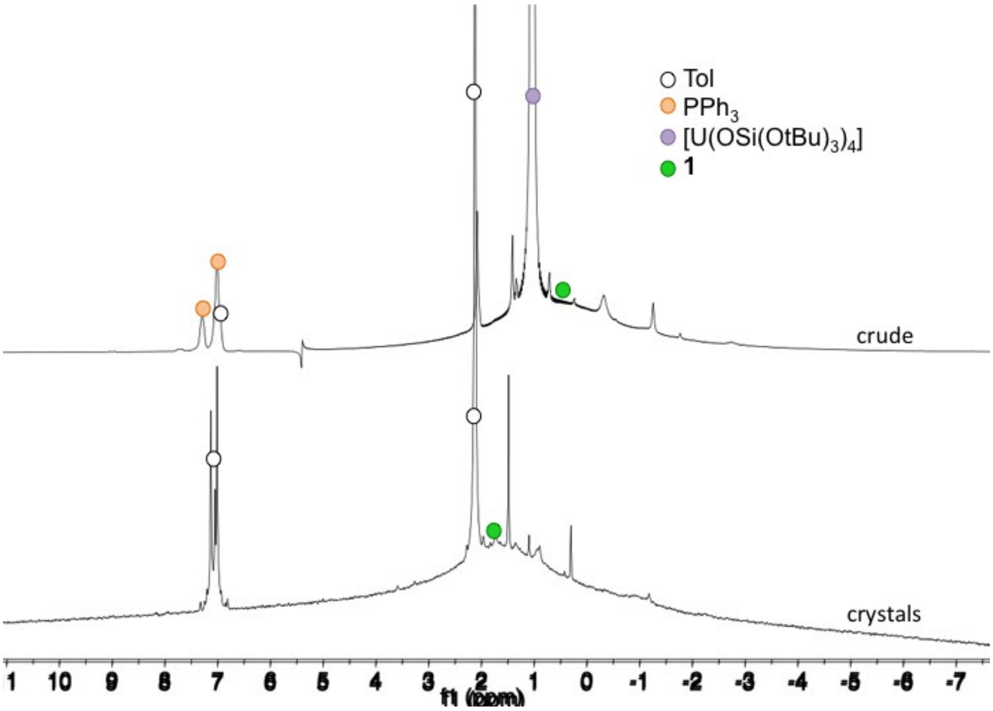

Figure S.B.2. Variable temperature ^{1}H NMR spectra of [US(OSi(OtBu)$_3$)$_4$K$_2$]$_2$ in anhydrous toluene-d_8 (400 MHz).

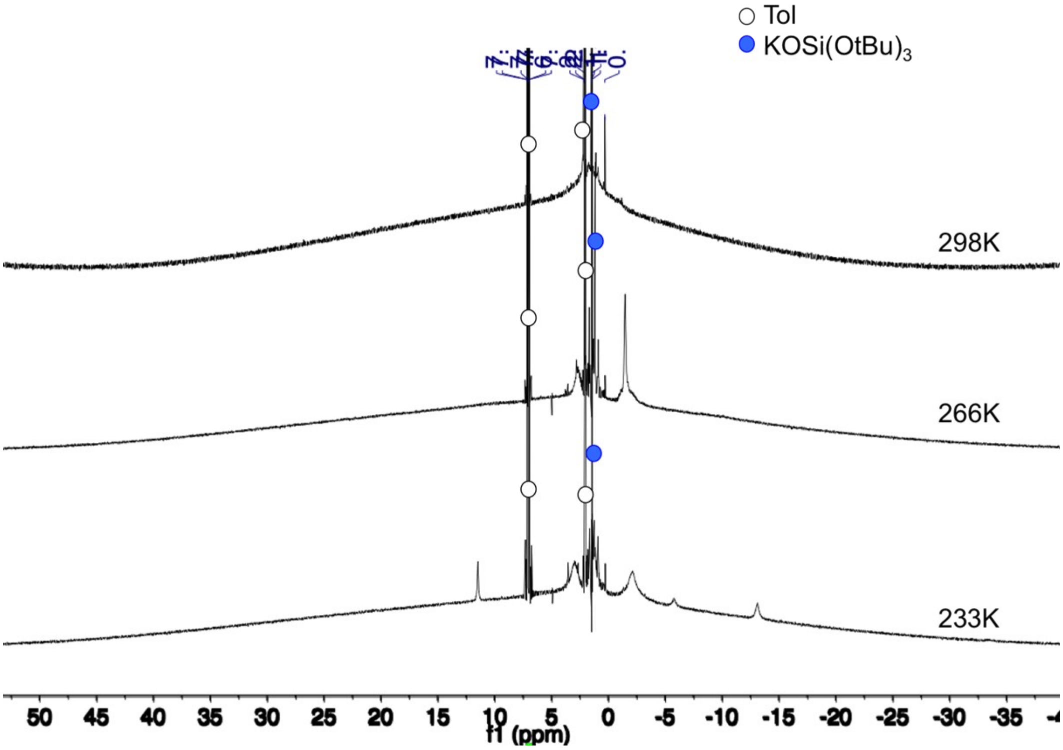

Figure S.B.3. ^{1}H NMR spectrum of the 1:1 reaction mixture of $[U(OSi(OtBu)_3)_4][K18c6]$ and Ph_3PS (bottom) and of $[\{US(OSi(OtBu)_3)_4K_2\}_2 18c6]$ **2** in anhydrous toluene-d_8 (200 MHz, 298 K).

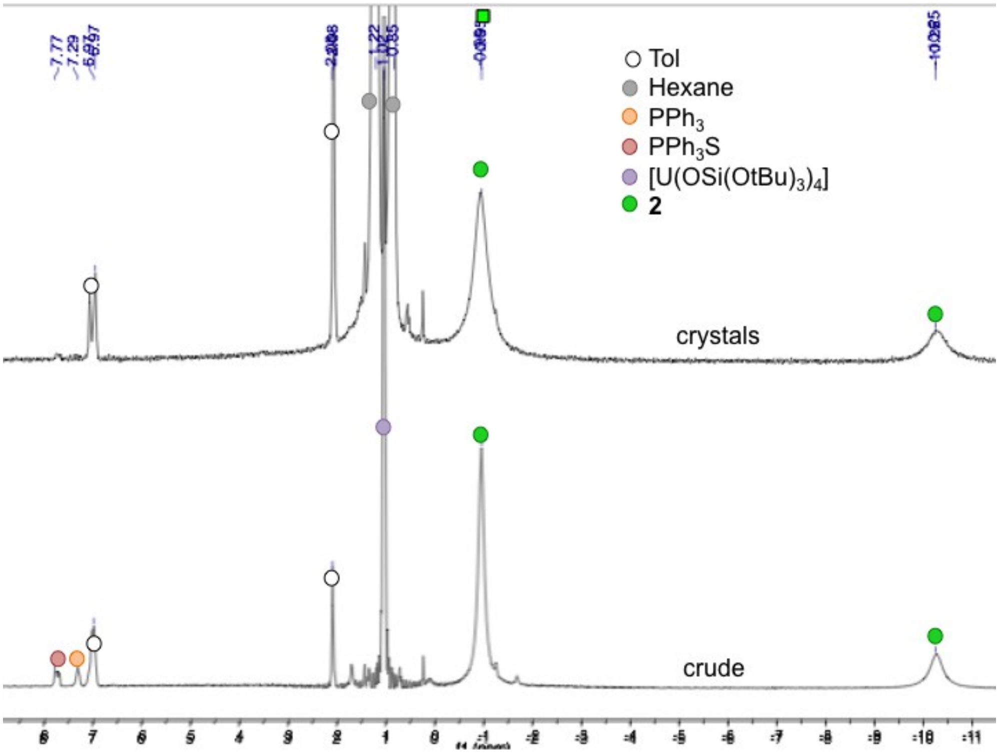

Figure S.B.4. ^{1}H NMR spectrum of the 1:1 reaction mixture of $[U(OSi(OtBu)_3)_4][K18c6]$ and Ph_3PS after 7 days in anhydrous THF-d_8 (200 MHz, 298 K).

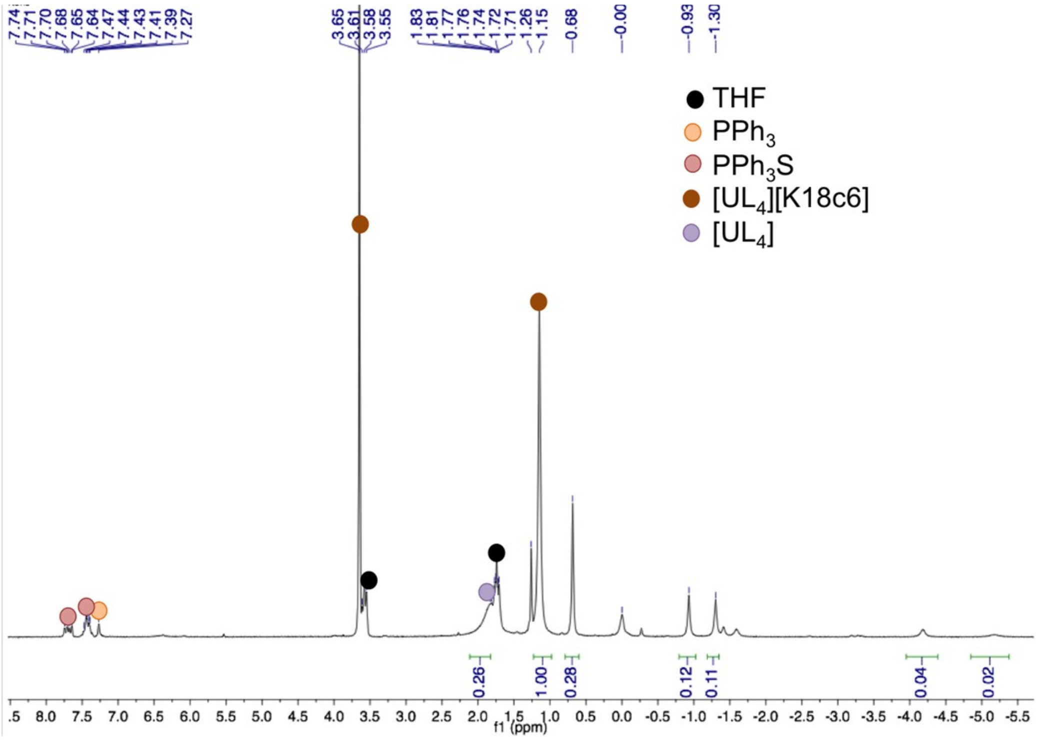

Figure S.B.5. ^{1}H NMR spectrum of the 1:1 reaction mixture of [U(OSi(OtBu)$_3$)$_4$K] and Ph_3PS (bottom), after addition of 1 eq. per U of 18c6 to the crude mixture (middle) and after another addition of 1 eq. per U of 18c6 in anhydrous toluene-d_8 (400 MHz, 298 K).

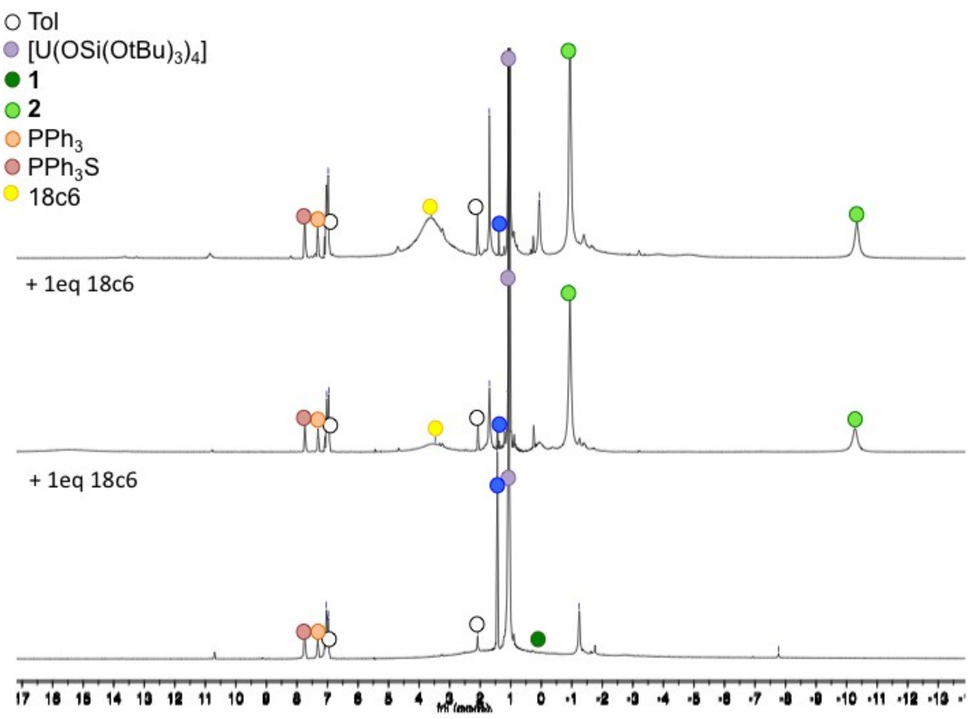

Figure S.B.6. ^{1}H NMR spectrum after addition of 1 eq. of cryptand per U atom to [US(OSi(OtBu)$_3$)$_4$K$_2$]$_2$ yielding complex **3** in anhydrous toluene-d_8. Stability of the complex was tested over 1 month(400 MHz, 298 K).

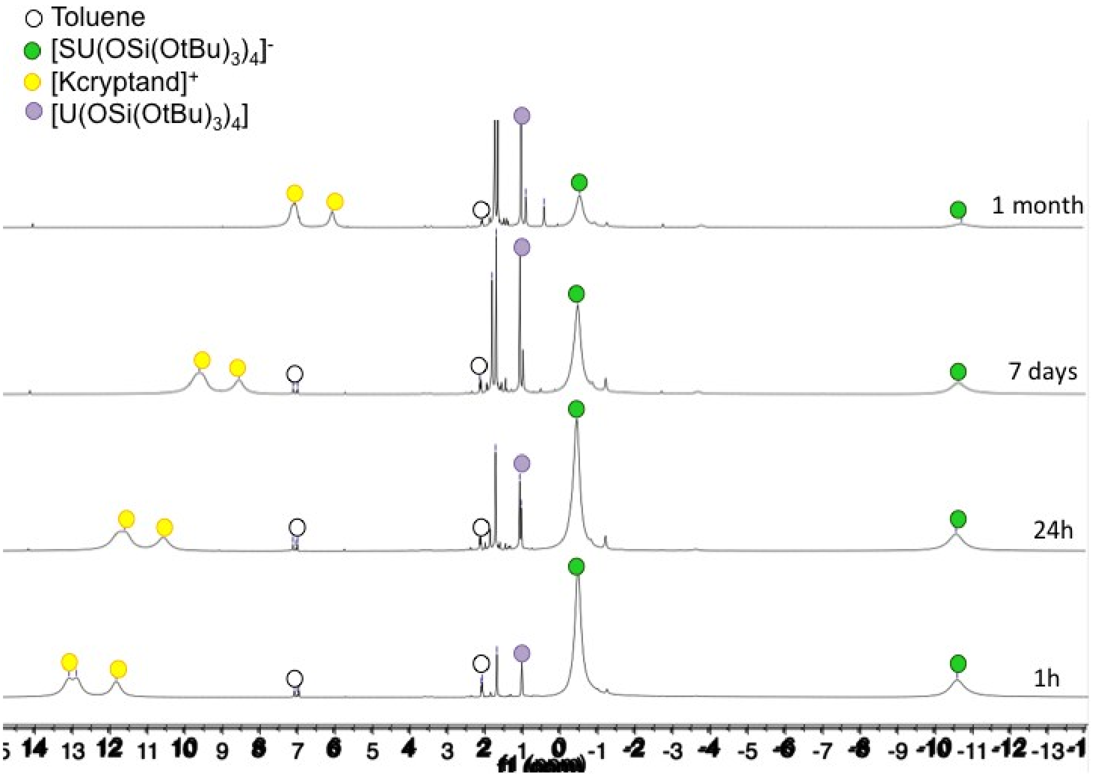

Figure S.B.7. ^{1}H NMR spectrum of the starting complex $[US(OSi(OtBu)_3)_4K_2]_2$ **1** (bottom) and after addition of 1 eq. of $^{13}CS_2$ per U atom to **1** in anhydrous toluene-d_8 (400 MHz, 298 K).

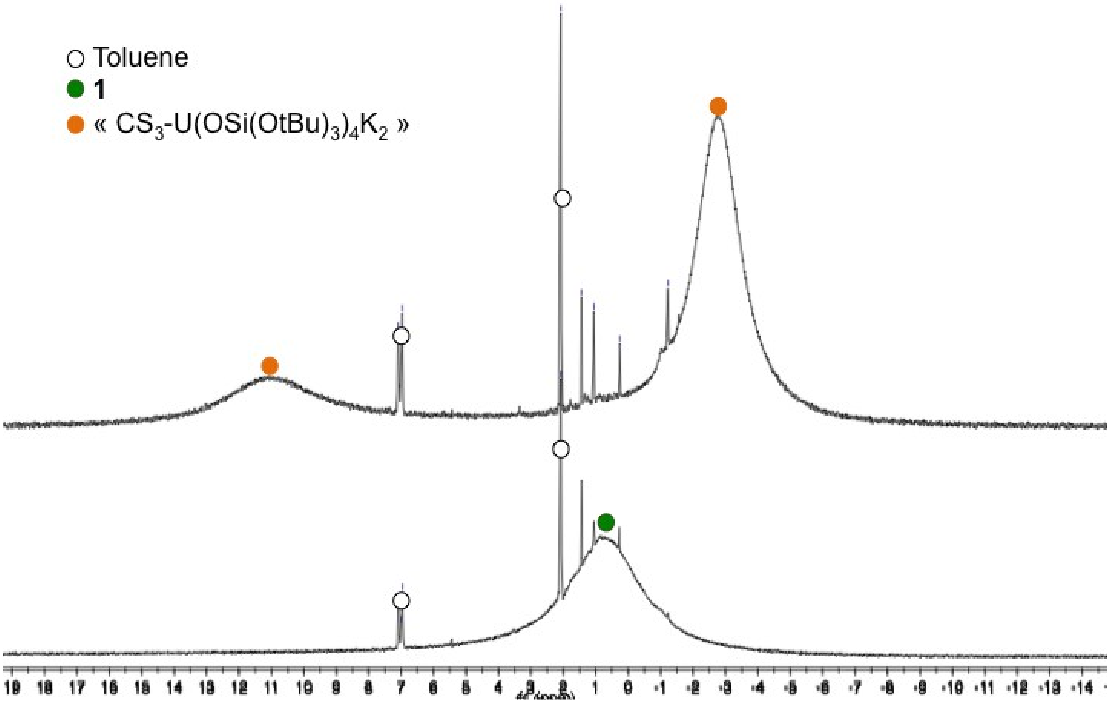

Figure S.B.8. ^{13}C NMR spectrum of the 1:2 reaction mixture of complex $[US(OSi(OtBu)_3)_4K_2]_2$ **1** and $^{13}CS_2$ in anhydrous DMSO-d_6 (400 MHz, 298K).

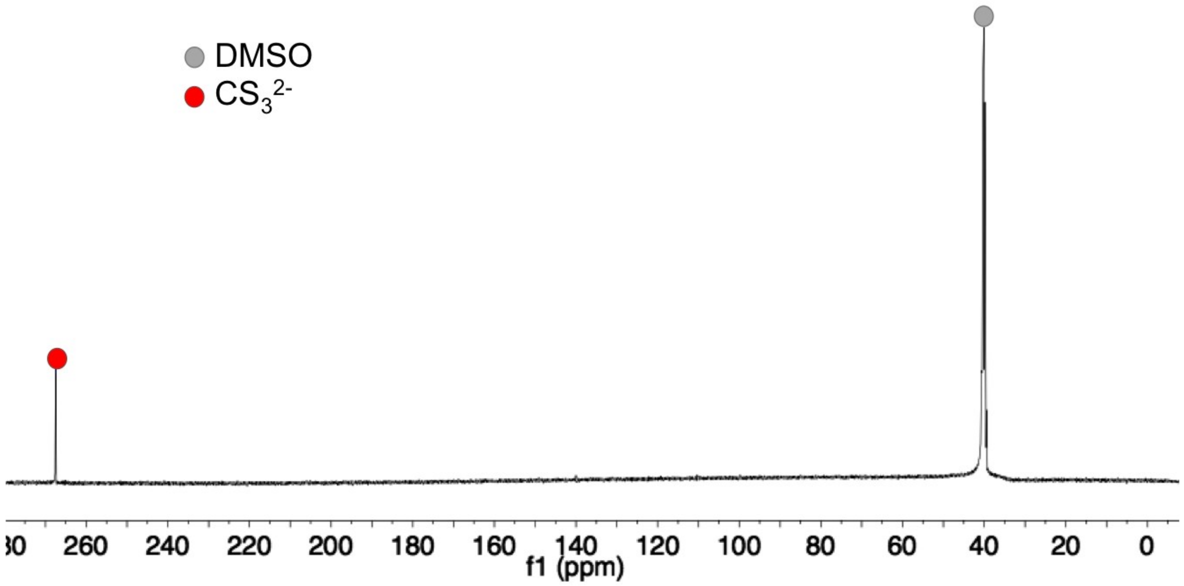

Figure S.B.9. ^{1}H NMR spectrum of the crude mixture after addition of an excess of $^{13}CO_2$ to $[US(OSi(OtBu)_3)_4K_2]_2$ **1** in anhydrous toluene-d_8 (400 MHz, 298 K).

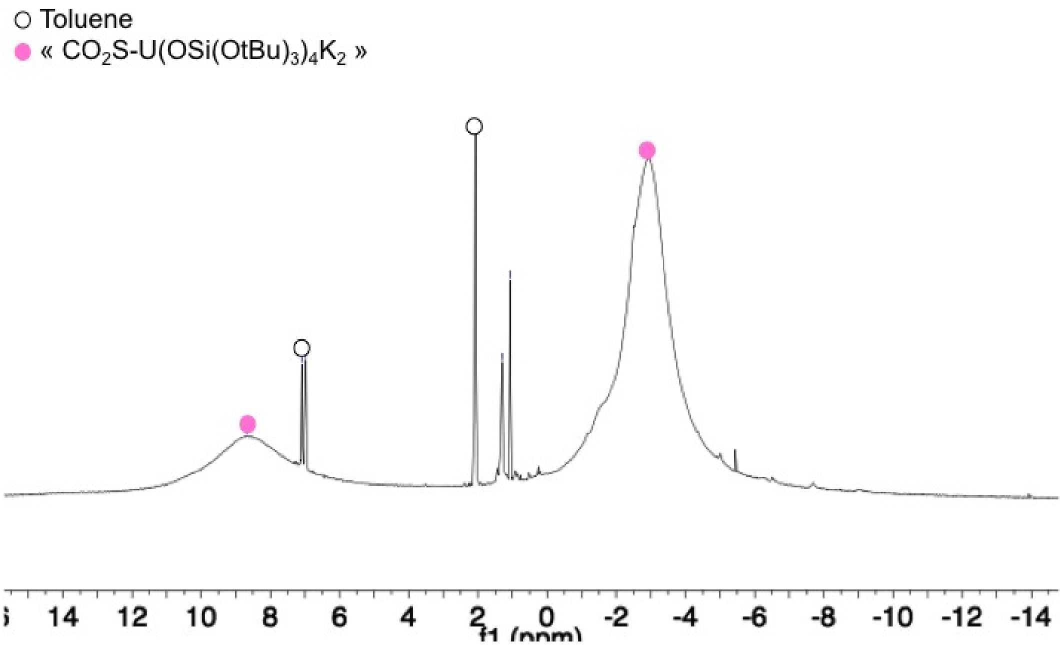

Figure S.B.10. ^{1}H NMR spectrum of the crude mixture after addition of an excess of $^{13}CO_2$ to $[US(OSi(OtBu)_3)_4K_2]_2$ **1** after 5 min (bottom) and after 18 h (top) in anhydrous toluene-d_8 (400 MHz, 298 K).

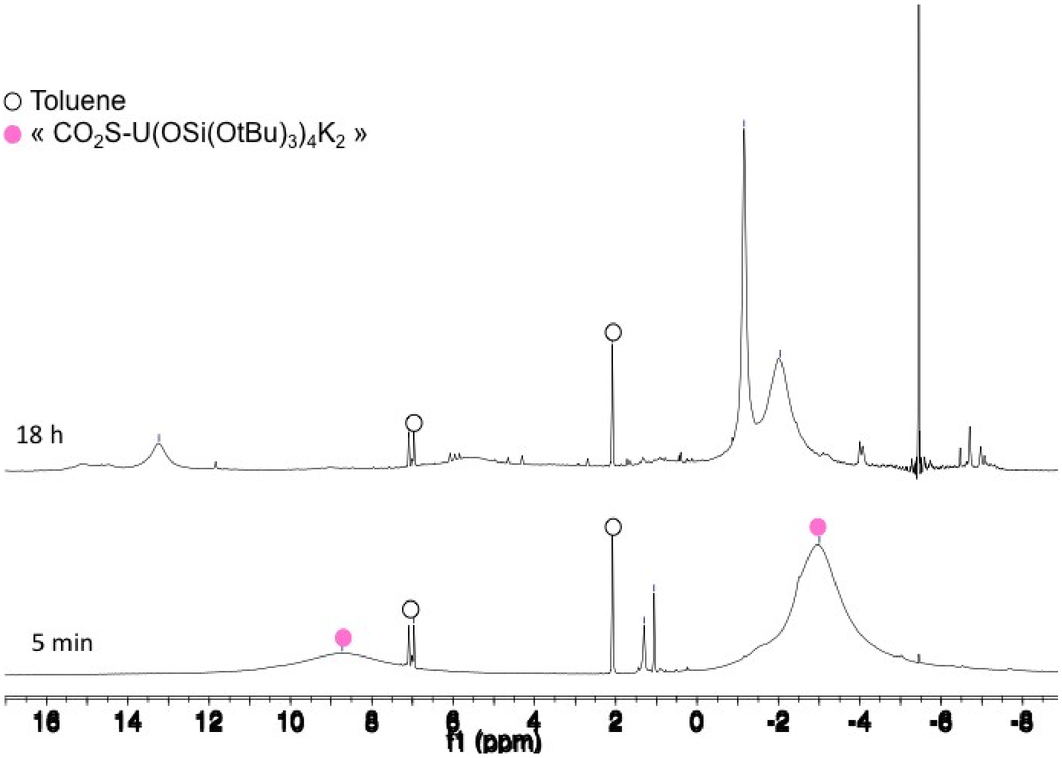

Figure S.B.11. ^{13}C NMR spectrum of the crude mixture after addition of an excess of CO_2 to $[US(OSi(OtBu)_3)_4K_2]_2$ **1** in anhydrous toluene-d_8 (400 MHz, 298 K).

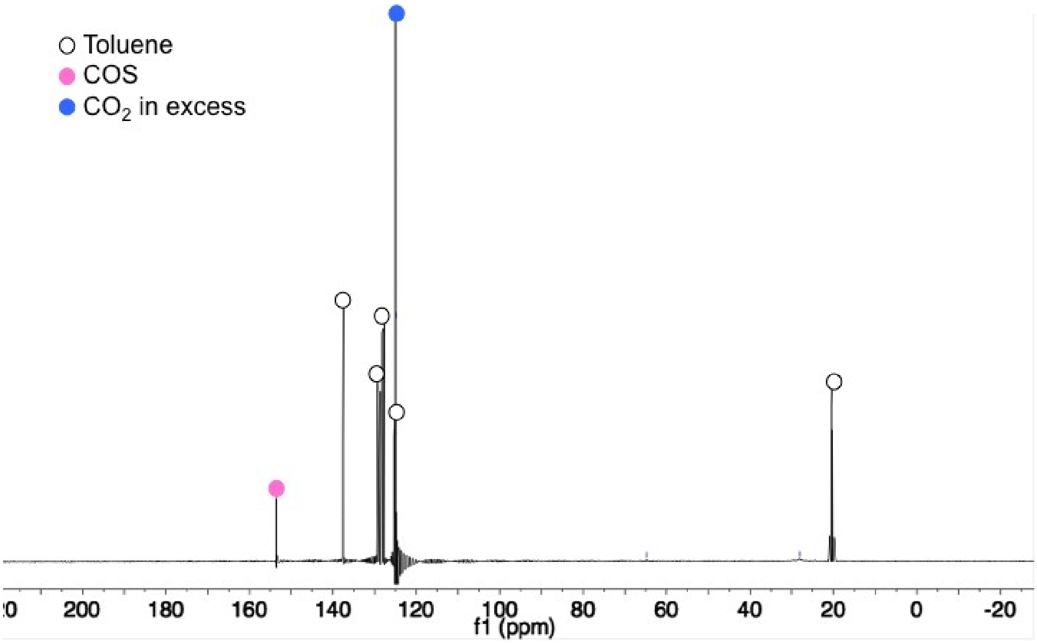

Figure S.B.12. ^1H NMR spectrum of the crude mixture after addition of 1 eq. of 18c6 and 1 eq. of PyHCl per U atom to $[US(OSi(OtBu)_3)_4K_2]_2$ **1** in anhydrous THF-d_8 (400 MHz, 298 K).

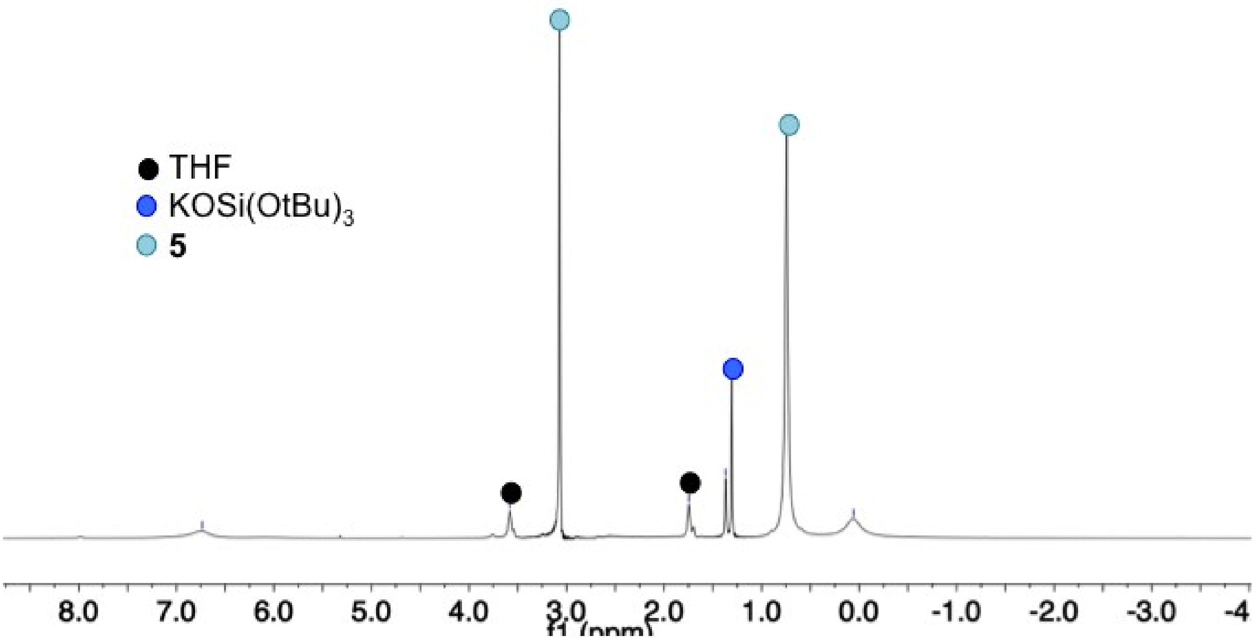

Figure S.B.13. ^{1}H NMR spectrum of complex **5** in anhydrous THF-d_8 (400 MHz, 298 K).

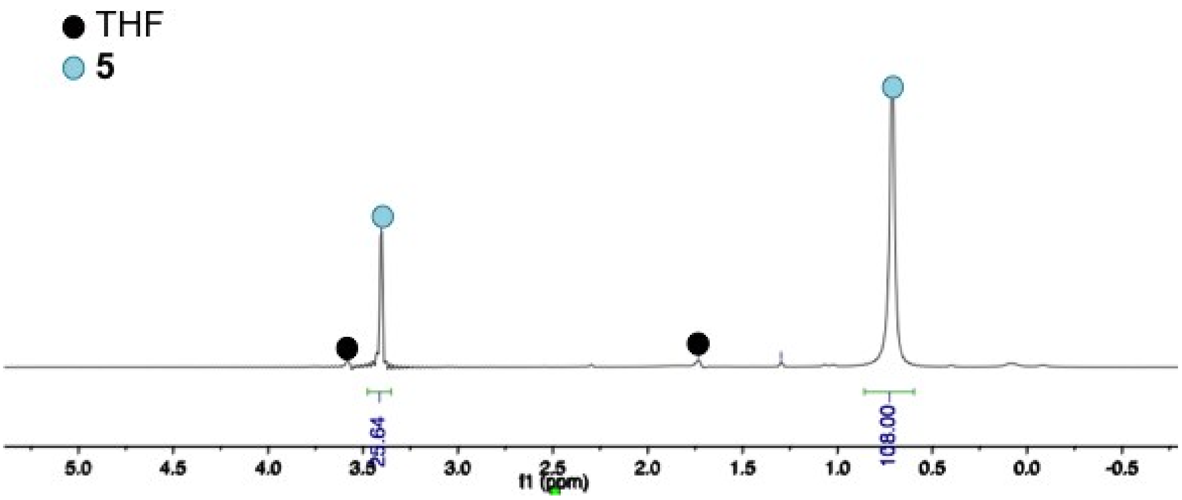

Figure S.B.14. ^{1}H NMR spectra of the stability follow up of complex **5** in anhydrous THF-d_8 (400 MHz, 298 K).

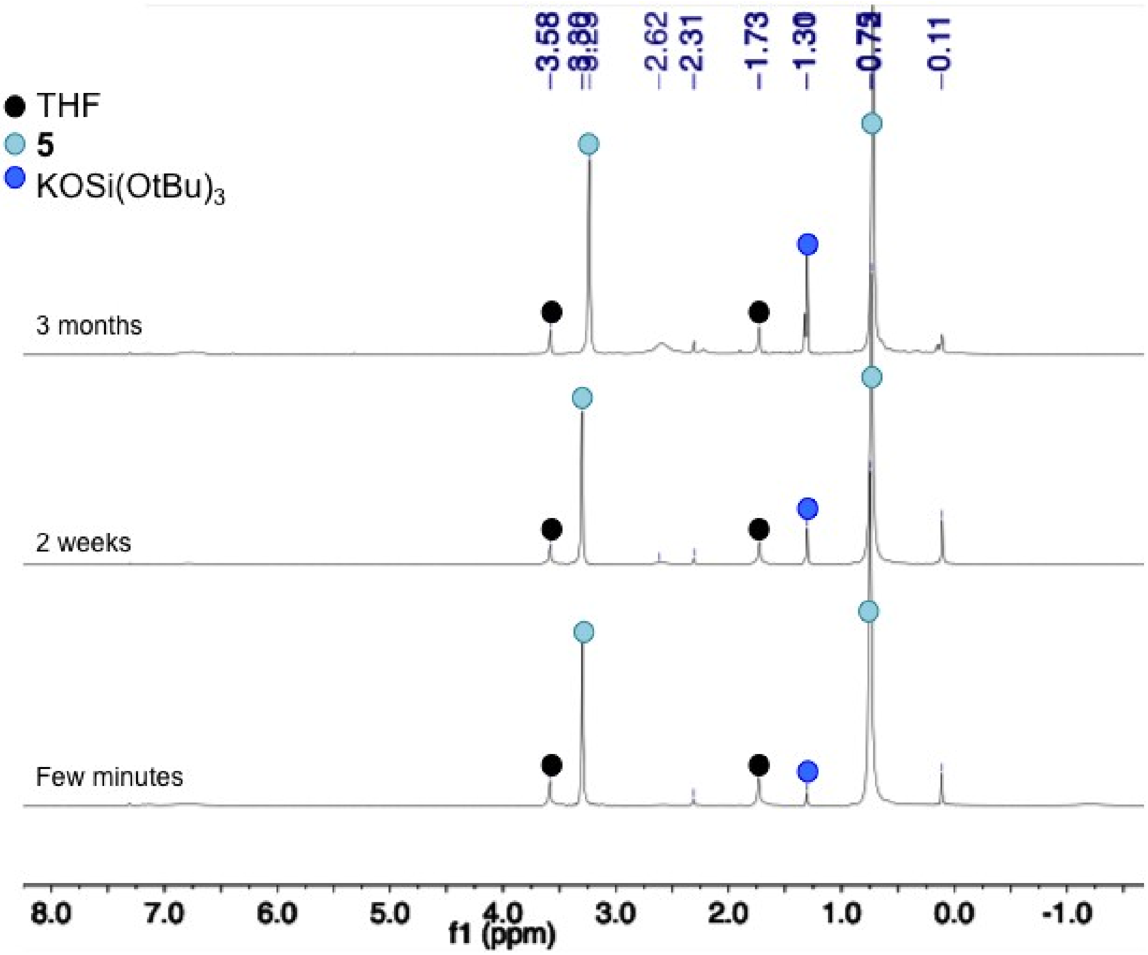

C) X-ray crystallographic data

Compound	**1 .tol**	**2.tol**	**3.hex**	**4**
Formula	$C_{103}H_{224}K_4O_{32}S_2Si_4U_2$	$C_{122}H_{256}K_4O_{38}S_2Si_8U_2$	$C_{72}H_{158}K_2N_2O_{22}SSi_4U$	$C_{90}H_{198}K_4N_2O_{30}S_3Si_6U_2$
Crystal size [mm]	0.80x0.70x0.60	0.216x0.095x0.020	0.39x0.26x0.21	0.231x0.177x0.063
Crystal system	Triclinic	Monoclinic	Triclinic	Orthorhombic
Space group	P -1	$P\ 2_1/n$	P-1	$P\ 2_12_12$
V [Å^3]	3592.8(3)	8250.6(11)	4919.2(13)	7250.0(3)
a [Å]	13.2622(7)	16.3304(14)	14.610(2)	27.6846(8)
b [Å]	15.3715(6)	26.8000(19)	17.221(3)	17.5786(4)
c [Å]	20.3762(10)	18.8582(15)	19.8684(19)	14.8975(4)
α [°]	76.530(4)	90	84.563(10)	90
β [°]	74.270(4)	91.476(9)	84.218(9)	90
γ [°]	65.093(4)	90	83.002(12)	90
Z	1	2	2	2
Absorption coefficient [mm^{-1}]	2.525	2.209	1.864	2.495
T [K]	150(2)	150(2)	100(2)	150(2)
Total no. reflexions	43383	37944	75038	43886
Unique reflexions [R(int)]	21655 [0.0499]	16850 [0.1343]	27810 [0.0677]	21867 [0.0851]
Final R indice [I>2 σ(I)]	0.0449	0.0778	0.0677	0.0752
Largest diff. peak and hole [eA^{-3}]	2.712 and -2.218	1.422 and -1.071	3.508 and -1.868	1.995 and -1.027
GOF	1.017	0.931	1.181	1.021

Compound	**5.tol**	**6.tol**
Formula	$C_{67}H_{141}KO_{22}SSi_4U$	$C_{79}H_{170}K_2O_{24}S_3Si_6U_2$
Crystal size [mm]	0.890x0.144x0.027	0.147x0.143x0.096
Crystal system	Monoclinic	Monoclinic
Space group	C c	$P\ 2_1$
V [Å^3]	8891.0(8)	5642.4(11)
a [Å]	13.8962(5)	13.9735(17)
b [Å]	24.5807(16)	18.2720(11)
c [Å]	26.1417(14)	22.168(3)
α [°]	90	90
β [°]	95.317(5)	94.534(12)
γ [°]	90	90
Z	4	2
Absorption coefficient [mm^{-1}]	2.011	3.117
T [K]	150(2)	100(2)
Total no. reflexions	20499	72530
Unique reflexions [R(int)]	11450 [0.0559]	22269 [0.0680]
Final R indice [I>2 σ(I)]	0.0674	0.0463
Largest diff. peak and hole [eA^{-3}]	1.676 and -1.648	2.307 and -1.485
GOF	1.075	1.156

Compound	**$[(S_2)U(OSi(OtBu)_3)_4K_2]_2$.tol**	**$\{[UK(OSi(OtBu)_3)_3]_2(\mu\text{-}S_2)(\mu\text{-}S_3)\}$.tol**
Formula	$C_{55}H_{116}K_2O_{16}S_2Si_4U$	$C_{79}H_{170}K_2O_{24}S_{4.36}Si_4U_2$
Crystal size [mm]	0.288x0.171x0.018	0.230x0.230x0.180
Crystal system	Orthorombic	Monoclinic
Space group	P bca	P 2_1/n
V [Å^3]	15151.7(6)	5644.5(6)
a [Å]	19.2882(5)	14.1455(8)
b [Å]	19.3780(5)	18.0590(13)
c [Å]	40.5378(9)	22.1126(12)
α [°]	90	90
β [°]	90	92.218(5)
γ [°]	90	90
Z	8	2
Absorption coefficient [mm^{-1}]	2.425	3.142
T [K]	150(2)	150(2)
Total no. reflexions	89393	34303
Unique reflexions [R(int)]	23090 [0.0718]	17040 [0.0446]
Final R indice [I>2 σ(I)]	0.0550	0.0475
Largest diff. peak and hole [eA^{-3}]	2.555 and -1.221	1.400 and -1.013
GOF	1.082	1.034

Complex $[(S_2)U(OSi(OtBu)_3)_4K_2]_2$.tol crystallizes in the orthorombic Pbca space group as a dimer. The solid state structure shows that two $[(S_2)U(OSi(OtBu)_3)_4K]$ moieties are bridged by two potassium cations capping the terminal persulfides to yield a dimer. Two potassium cations (each one also bound to a OtBu group) and the two terminal persulfides form a S_2KS_2K diamond core around the inversion center. The second potassium ion of the asymmetric unit is located in an O6 coordination pocket formed by three siloxide ligands.

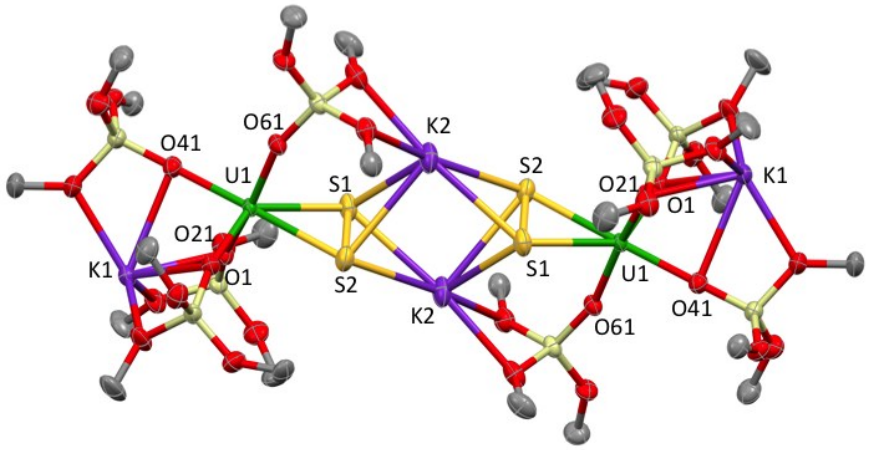

Figure S.C.1: Ellipsoid diagram of complex $[(S_2)U(OSi(OtBu)_3)_4K_2]_2$.tol (50% probability ellipsoids). Methyl groups and solvent molecules are omitted for clarity.

The {[UK(OSi(OtBu)$_3$)$_3$]$_2$(**μ**-S$_2$)(**μ**-S$_3$)}**.**tol complex crystallizes in the Monoclinic P 2$_1$/n space group as a dimer. The solid state structure shows that two [U(OSi(OtBu)$_3$)$_3$K] moieties are bridged by disordered S_3^{2-} and S_2^{2-} ligands (occupancy factor of 0.183(5) and 0.817(5) respectively). The potassium ion of the asymmetric unit is located in an O6 coordination pocket formed by three siloxide ligands.

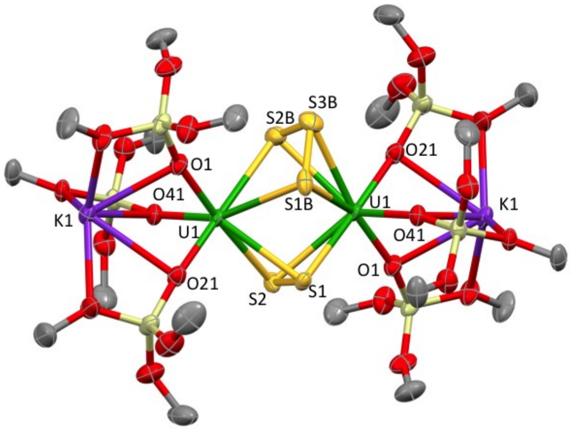

Figure S.C.2: Ellipsoid diagram of complex {[UK(OSi(OtBu)$_3$)$_3$]$_2$(**μ**-S$_2$)(**μ**-S$_3$)}**.**tol (50% probability ellipsoids). Methyl groups, disorder and solvent molecules are omitted for clarity.

Complex **4** crystallizes in the orthorhombic space group, P2$_1$2$_1$2, as an ion pair consisting of the [U$_2$(μ-S)$_3$(OSi(OtBu)$_3$)$_6$K$_3$]$^-$ anion and the [Kcryptand]$^+$ cation. In the [U$_2$(μ-S)$_3$(OSi(OtBu)$_3$)$_6$K$_3$]$^-$ anion, three S^{2-} anions bridge the two uranium atoms. Each uranium(IV) ion is six-coordinate by three oxygen atoms from three siloxide ligands, and three sulfide anions in a distorted octahedral fashion. The average value of the U-S bond lengths is 2.748(7) Å, which is in agreement with the values of U-S bond lengths found in reported sulfide-bridged di-uranium(IV) complexes. The average U-O bond length (2.258(3) Å) is in the range of reported bond lengths for siloxide compounds. The average value for the S-K bond lengths (3.053(4) Å) is similar to the S-K bond length in complex **1** (3.0455(12) Å).

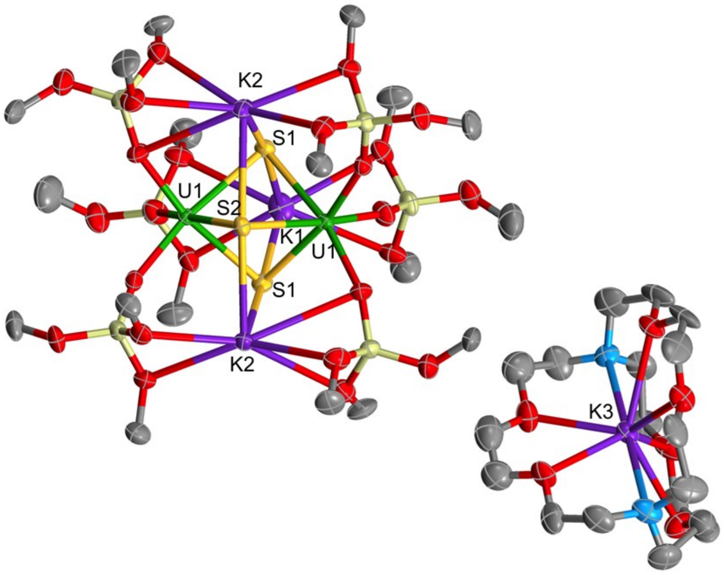

Figure S.C.3: Ellipsoid diagram of complex **4** [S$_3$U$_2$(OSi(OtBu)$_3$)$_6$K$_3$][Kcryptand] (50% probability ellipsoids). Methyl groups are omitted for clarity.

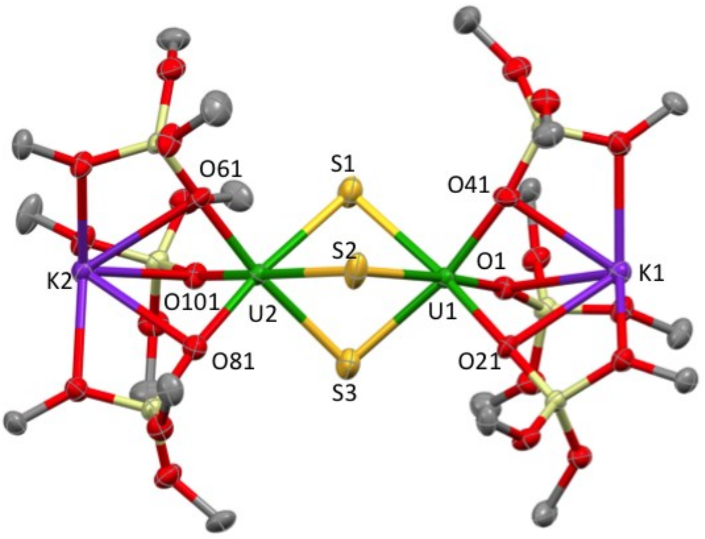

Figure S.C.4: Ellipsoid diagram of complex **6.**tol (50% probability ellipsoids). Methyl groups, disorder and solvent molecules are omitted for clarity.

D) Computational results

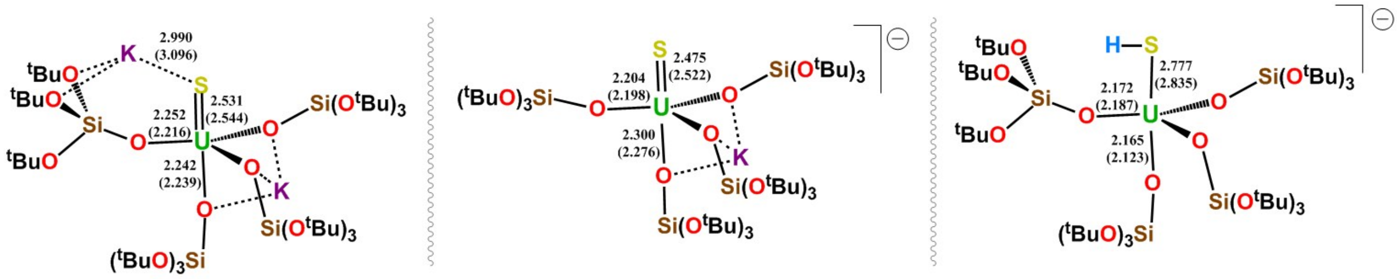

monomer-1 **complex-3** complex **5**

Figure S.D.1. Selected bond distances (in angstrom) of the optimized and X-ray crystal structures. The experimental distances are given in parenthesis.

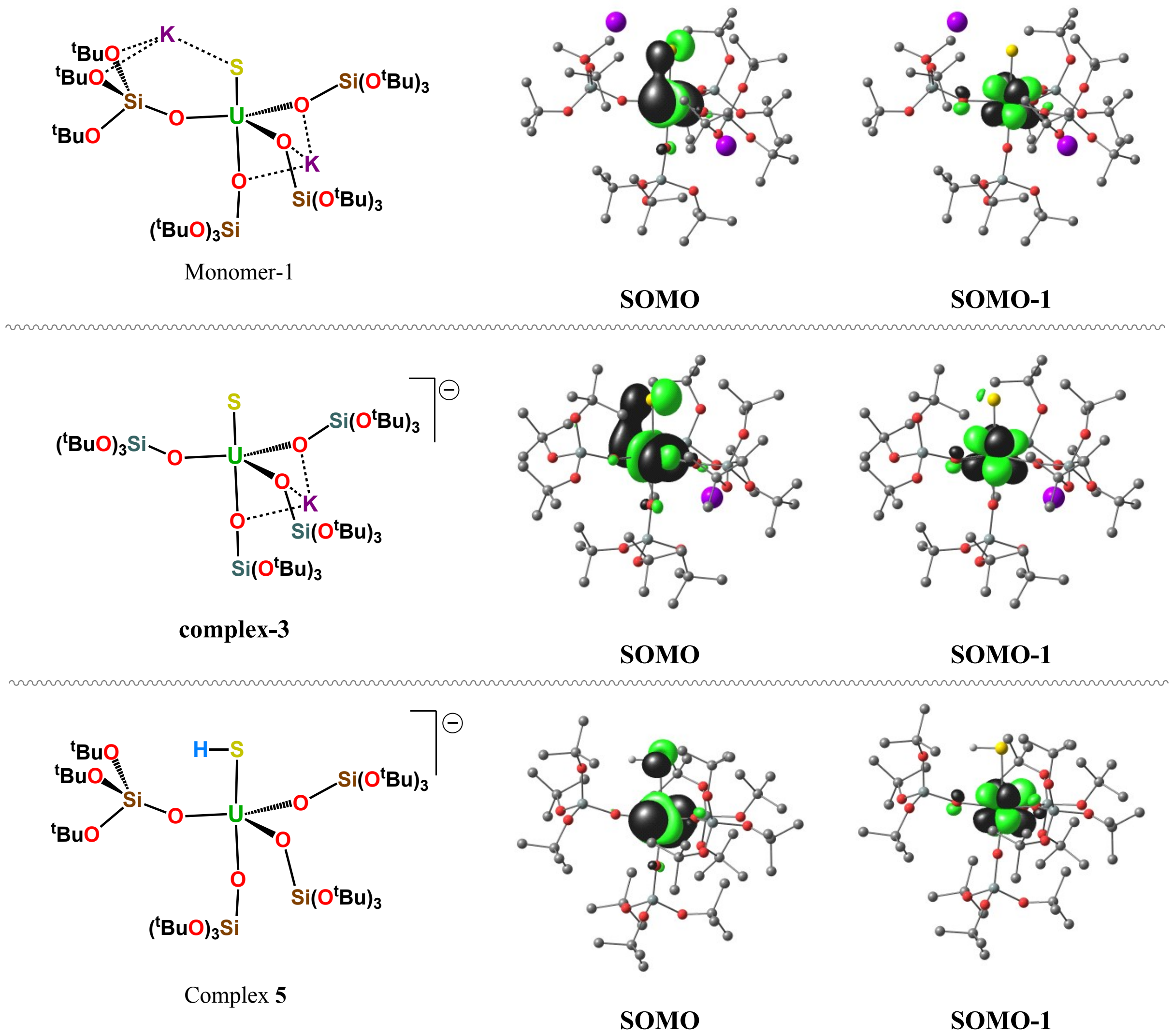

Figure S.D.2 The SOMOs (Single Occupied Molecular Orbitals) for the **monomer-1**, **3**, and **5** model complexes.

Cartesian coordinates of the optimized structures.

monomer-1 (triplet, small-core)

U	14.55343400	5.07707900	4.05901200
K	12.61098600	2.25982400	5.08920900
K	17.09816100	7.93614700	1.40208900
S	16.07049700	5.20062000	2.03646400
Si	15.63989900	8.68403900	4.31784800
Si	11.72133600	3.68197600	1.94991200
Si	12.41578500	5.04170500	7.26501500
Si	16.08323400	1.68177500	4.98505100
O	14.89027200	7.25972600	4.49817700
O	17.31233300	8.59501400	4.04829100
O	15.26315700	9.65189900	5.62203200
O	15.29680000	9.44939700	2.83981500
O	12.71035800	4.39636700	3.03873400
O	11.21447900	2.26676300	2.74250800
O	10.41270800	4.62058600	1.49399600
O	12.39974300	3.15075000	0.53070600
O	13.43508400	4.93215800	5.99673400
O	11.59494100	3.55261100	7.25040300
O	11.20967400	6.18642400	7.15754100
O	13.14841300	5.31698700	8.74360000
O	15.31826400	3.08210200	4.63811300
O	14.79677700	0.63916400	5.37528900
O	17.03320300	1.71409900	6.36118800
O	17.07442200	1.05361300	3.80641800
C	18.33391100	7.95407100	4.86322500
C	17.74498700	7.27147400	6.10395200
H	17.06880300	6.46636500	5.80543200
H	17.18399600	7.98977300	6.71082300
H	18.55475700	6.84757500	6.70828800
C	19.03674700	6.91545800	3.97504600
H	19.80121800	6.38108300	4.55011900
H	19.54137800	7.41829700	3.13620500
H	18.30872200	6.18874700	3.59041500
C	19.33693100	9.04049600	5.28113800
H	18.86546600	9.75597600	5.96140400
H	19.69527600	9.57702100	4.39466100
H	20.19366600	8.58356500	5.78993000
C	15.62446200	11.00509000	5.98842100
C	16.55299500	11.68438600	4.96963800
H	16.78538400	12.69913400	5.31403100
H	16.06764100	11.74234700	3.99107300
H	17.48348400	11.12152200	4.86221200
C	14.31656200	11.80240900	6.11691300
H	14.52221400	12.80795800	6.50188100
H	13.63807100	11.28532900	6.80290200
H	13.83124400	11.89179700	5.13944100
C	16.30646100	10.91950700	7.36318800
H	17.23747800	10.34923400	7.28458600
H	15.64134500	10.41206200	8.07006000
H	16.53575600	11.92322000	7.73946200
C	13.98557200	9.73255100	2.27967800
C	12.84755100	9.47814600	3.27758800
H	12.82713200	8.42524300	3.57016100
H	11.89043400	9.73625200	2.81044400
H	12.98153600	10.08718500	4.17574100
C	13.99858900	11.20958000	1.85726100
H	13.06270900	11.46676700	1.34796500
H	14.83750400	11.39647400	1.17602200
H	14.11170200	11.85079900	2.73749900
C	13.80782100	8.83311400	1.04704300
H	13.90972400	7.77989300	1.33206900
H	14.55814000	9.08198000	0.28241700
H	12.81769100	8.98386100	0.60292100
C	10.50358600	1.14118800	2.16140000
C	9.44064700	1.59271100	1.14884700
H	8.89563700	0.71573100	0.77945200
H	9.91184200	2.10026600	0.30297900
H	8.73263000	2.28067700	1.62092500
C	9.82059700	0.42843900	3.34066900
H	9.14186600	1.12005200	3.85248500
H	10.57326400	0.06687400	4.05293700
H	9.24854000	-0.43449100	2.98194600
C	11.51790700	0.20232700	1.49234100
H	12.25763500	-0.13329300	2.22839000
H	12.03792400	0.73770100	0.69372300
H	11.00594800	-0.67536700	1.07987200

C	9.58111100	5.47883300	2.30843700
C	8.26394100	5.62553500	1.53169600
H	8.46855000	6.02934400	0.53401300
H	7.58207200	6.30093700	2.06121200
H	7.78667800	4.64546100	1.42281700
C	9.31739400	4.86581500	3.69035500
H	8.89038300	3.86378800	3.57756200
H	8.61375600	5.49484300	4.24717200
H	10.24623100	4.80196300	4.26619400
C	10.27225600	6.84003800	2.45029500
H	11.22853100	6.70763000	2.96366700
H	9.64546700	7.52901900	3.02861400
H	10.45634800	7.26620600	1.45765900
C	14.38488600	4.77105800	9.26139600
C	14.28710600	4.92216200	10.78714800
H	14.13306500	5.97638100	11.04293300
H	15.20783200	4.56696000	11.26420100
H	13.43913800	4.34026600	11.16465800
C	13.58616500	-1.39239800	5.67710500
C	11.51397600	3.77412600	-1.62793800
C	17.87205600	1.86154100	1.68236900
H	17.57168600	2.89456400	1.89367300
H	17.84678100	1.70629600	0.59675700
H	18.89220800	1.68341800	2.04355000
C	12.71495200	3.89205500	-0.67902100
C	13.03299000	5.36071800	-0.37444300
H	12.14968500	5.86221500	0.03485800
H	13.32956900	5.86752700	-1.30202600
H	13.86245500	5.41177400	0.34256600
C	13.95208200	3.20431800	-1.27167900
H	14.21922300	3.66922200	-2.22822800
H	13.74157700	2.14141100	-1.43745000
H	14.78943000	3.30530400	-0.57313700
C	10.43498700	3.16815000	8.03443600
C	10.42688700	1.62991100	8.03443800
H	10.32072100	1.25089500	7.00976500
H	9.58315100	1.25509400	8.62463500
H	11.36030500	1.25112600	8.46631900
C	9.16751900	3.70081500	7.35096600
H	9.21133700	4.79199500	7.30550100
H	8.27684900	3.38721400	7.90860200
H	9.10276900	3.30971600	6.32941700
C	10.51880200	3.67960600	9.48041300
H	11.43095400	3.31410200	9.96167700
H	9.64912300	3.31893200	10.04282000
H	10.52574200	4.77254000	9.50079900
C	11.23277200	7.58158100	7.55732200
C	10.20036100	8.27837400	6.65902000
H	10.50745900	8.19443200	5.61140000
H	10.11814400	9.33809600	6.92729300
H	9.22151000	7.80075000	6.78084700
C	10.80409500	7.66962800	9.02962400
H	9.81023600	7.22430400	9.15601600
H	10.76542800	8.71782500	9.34984000
H	11.52489400	7.12820000	9.65056200
C	12.61863800	8.20437600	7.36110800
H	13.34225500	7.73273000	8.03252900
H	12.57542000	9.27652600	7.58585900
H	12.96596100	8.07757200	6.33223200
C	14.54762500	3.29347700	8.88280200
H	13.66958400	2.72702500	9.21009700
H	15.43925600	2.88055900	9.36725000
H	14.66922900	3.18169200	7.80079000
C	15.55577100	5.59985000	8.71889600
H	16.50538700	5.22338800	9.11742800
H	15.43531800	6.64966500	9.00852900
H	15.57300900	5.53545300	7.62768000
C	14.85650900	-0.62671600	6.08308200
C	16.09539500	-1.44649300	5.69845500
H	16.10402400	-1.63983300	4.62251000
H	16.07855100	-2.40350600	6.23366600
H	17.00590800	-0.90429500	5.96601100
C	14.84653400	-0.34775000	7.59312100
H	14.84725800	-1.29054000	8.15319500
H	13.94945100	0.22239400	7.86138200
H	15.72910400	0.24027000	7.85799400
C	18.41899800	2.12121500	6.48381200
C	19.32618900	0.93649200	6.11929400

H	19.10330300	0.08545200	6.77352200
H	20.37932200	1.21395000	6.24849900
H	19.14817500	0.64769500	5.07959800
C	18.60347200	2.49490800	7.96233900
H	18.35413400	1.63489800	8.59426500
H	17.93859000	3.32664600	8.21794300
H	19.64191000	2.78969200	8.15350900
C	18.72219700	3.33101500	5.59058300
H	19.75696100	3.65872100	5.74895900
H	18.04607800	4.15478000	5.83886500
H	18.58895400	3.07360200	4.53531500
C	16.90525000	0.89043300	2.37117000
C	17.29821500	-0.56175500	2.06064700
H	18.31240900	-0.75758500	2.42618800
H	17.26666700	-0.73534800	0.97882000
H	16.60530800	-1.25355400	2.55138600
C	15.46397000	1.16444200	1.93240300
H	14.77274000	0.51477100	2.48255500
H	15.35883200	0.95915400	0.86135800
H	15.20860900	2.21439700	2.10518900
H	12.69122300	-0.83348800	5.97979100
H	13.56000400	-2.37120400	6.16902400
H	13.56878900	-1.53728700	4.59111100
H	11.30602400	2.71757800	-1.83429000
H	11.72859200	4.28390800	-2.57480500
H	10.63435200	4.23055500	-1.16206300

monomer-1 (singlet, large-core)

U	14.56792600	5.07969000	3.93613700
K	12.59519300	2.27387900	5.12169200
K	17.15010200	8.04595000	1.38246400
S	16.06067600	5.30447200	1.79834500
Si	15.69558900	8.68834000	4.35317100
Si	11.66352500	3.68480200	1.91755300
Si	12.39211500	5.02200800	7.24812400
Si	16.10859000	1.67883700	4.95555200
O	14.96638200	7.25653300	4.54032800
O	17.36509300	8.61118800	4.05729800
O	15.33564700	9.64180400	5.67159100
O	15.32577400	9.46270500	2.88716400
O	12.65760400	4.43666200	2.97746200
O	11.19355300	2.28324900	2.75240500
O	10.34171200	4.60350600	1.46672600
O	12.33794400	3.13550300	0.50584800
O	13.39879800	4.87743700	5.98751400
O	11.54882000	3.53873000	7.27163000
O	11.18714800	6.17495900	7.13282000
O	13.10969600	5.31446600	8.73696600
O	15.36314300	3.07266400	4.55111300
O	14.80756100	0.65585200	5.34027200
O	17.02673600	1.74311600	6.35154400
O	17.12520100	1.02432500	3.81445800
C	18.39894300	7.96120500	4.84742800
C	17.83806800	7.29159200	6.10882700
H	17.14877500	6.48720000	5.84125100
H	17.29640900	8.01860500	6.72228700
H	18.66133600	6.87008200	6.69640100
C	19.06887500	6.91441100	3.94317300
H	19.82602800	6.35542400	4.50427400
H	19.57668400	7.41226600	3.10374100
H	18.32193400	6.21065400	3.55278900
C	19.42223800	9.03947400	5.23771200
H	18.97379200	9.76019000	5.92830700
H	19.76186900	9.57187300	4.34148000
H	20.28827400	8.57666700	5.72489800
C	15.69348900	10.99485600	6.04203200
C	16.60435400	11.68641900	5.01561400
H	16.83398400	12.70081500	5.36279400
H	16.10671200	11.74644200	4.04349400
H	17.53739900	11.13050900	4.89414900
C	14.38159600	11.78115700	6.19333600
H	14.58410700	12.78715800	6.57874600
H	13.71757300	11.25561600	6.88701200
H	13.88116100	11.86916500	5.22332900
C	16.39614500	10.90376500	7.40583700
H	17.33155000	10.34327800	7.30797000
H	15.74658500	10.38374800	8.11795600

H	16.62161300	11.90627000	7.78753600
C	14.00961200	9.75654700	2.34360600
C	12.87793800	9.44717800	3.33322300
H	12.86081200	8.38125400	3.57308400
H	11.91742500	9.72603800	2.88539300
H	13.01464800	10.00845400	4.26199400
C	14.00196600	11.25059900	1.98516700
H	13.06365200	11.51488800	1.48401700
H	14.83939700	11.47810400	1.31464200
H	14.10249900	11.85594500	2.89166600
C	13.83967600	8.91144200	1.07141900
H	13.97730800	7.84819600	1.30064300
H	14.57322000	9.21693600	0.31115300
H	12.84017600	9.05628800	0.64667400
C	10.48915400	1.13618400	2.20359500
C	9.40566100	1.55461000	1.19869500
H	8.86383200	0.66430400	0.85758700
H	9.85768700	2.04766400	0.33388700
H	8.69846100	2.24594500	1.66707200
C	9.83252800	0.43830000	3.40642400
H	9.15727400	1.13302000	3.91851500
H	10.59955400	0.09280300	4.11125800
H	9.26106500	-0.43467600	3.07191500
C	11.50749300	0.19921300	1.53868100
H	12.26496600	-0.10648200	2.26968400
H	12.00500400	0.72454100	0.71934500
H	11.00377200	-0.69619600	1.15525700
C	9.51911400	5.47589500	2.27695400
C	8.19061600	5.60204400	1.51619800
H	8.37920200	5.98830400	0.50848600
H	7.51306300	6.28396500	2.04280900
H	7.71658800	4.61802700	1.43221000
C	9.27915800	4.88963700	3.67449900
H	8.85493300	3.88379900	3.58813100
H	8.58046100	5.52600800	4.22908300
H	10.21598000	4.84122400	4.23872000
C	10.20634600	6.84311200	2.37965900
H	11.16773200	6.72701000	2.88740600
H	9.58186400	7.54248500	2.94790600
H	10.37902300	7.24676500	1.37565300
C	14.34588800	4.78054200	9.26062500
C	14.24769200	4.94530500	10.78537300
H	14.08621500	6.00096200	11.03057200
H	15.17062100	4.60088200	11.26628900
H	13.40326000	4.36172500	11.16818200
C	13.57534600	-1.36440200	5.63386800
C	11.43380100	3.68343000	-1.66393200
C	17.85872900	1.93355200	1.70026300
H	17.44054800	2.92875900	1.89411300
H	17.87468800	1.77229000	0.61538200
H	18.88568600	1.87821400	2.08148800
C	12.63414700	3.85663200	-0.72322100
C	12.91494200	5.33982900	-0.45435000
H	12.02187400	5.82735600	-0.04922900
H	13.19041500	5.83195100	-1.39596400
H	13.75270700	5.43042400	0.24961900
C	13.88763600	3.18539300	-1.29933000
H	14.14228700	3.63328300	-2.26729500
H	13.70352200	2.11380500	-1.43860900
H	14.72313100	3.32711900	-0.60541000
C	10.38498000	3.17924000	8.05637500
C	10.35686800	1.64078800	8.07471600
H	10.25350600	1.25208200	7.05332600
H	9.50513500	1.28260200	8.66387000
H	11.28333100	1.25632300	8.51667700
C	9.12400700	3.71862500	7.36505900
H	9.18289900	4.80837800	7.30531600
H	8.22840900	3.42373800	7.92511900
H	9.05603700	3.31486500	6.34840100
C	10.47255000	3.70578800	9.49682700
H	11.38009300	3.33456300	9.98261400
H	9.59793400	3.36293000	10.06284200
H	10.49491500	4.79847200	9.50358200
C	11.21424800	7.57080200	7.52071900
C	10.20884000	8.27139500	6.59411300
H	10.53871800	8.17776100	5.55409500
H	10.12876600	9.33357500	6.85350900
H	9.22377100	7.80204800	6.69654900

C	10.75219200	7.67650500	8.98218700
H	9.75182500	7.24082000	9.08923400
H	10.71604600	8.72762900	9.29323800
H	11.45374400	7.13341100	9.62339900
C	12.60929400	8.18407900	7.35522200
H	13.31016500	7.71485100	8.05158400
H	12.56626900	9.25910900	7.56651400
H	12.98444200	8.04227400	6.33798900
C	14.51792700	3.29913000	8.89771100
H	13.63835300	2.73362200	9.22256400
H	15.40675200	2.89335200	9.39348000
H	14.64721100	3.18021300	7.81737000
C	15.51568400	5.60826100	8.71181900
H	16.46726200	5.23510300	9.10918700
H	15.39462700	6.65947300	8.99617300
H	15.52623300	5.53875600	7.62075400
C	14.85471700	-0.61517300	6.04170600
C	16.08341900	-1.44517000	5.64663000
H	16.09053000	-1.62267300	4.56769000
H	16.05400400	-2.40932800	6.16816700
H	17.00149200	-0.91935100	5.92144900
C	14.85143000	-0.34568600	7.55329200
H	14.84544500	-1.29220200	8.10695500
H	13.96013600	0.23020500	7.82855800
H	15.73914000	0.23335200	7.82044500
C	18.41256900	2.14201500	6.49622300
C	19.31959400	0.94694200	6.16739300
H	19.07875600	0.10808300	6.83087300
H	20.37169200	1.22023100	6.31257700
H	19.15969600	0.64223700	5.12938200
C	18.56986000	2.53673500	7.97229700
H	18.30598100	1.68637100	8.61112800
H	17.90226700	3.37322500	8.20377300
H	19.60540700	2.83116100	8.17898900
C	18.74002200	3.33745700	5.59149600
H	19.77303500	3.66282100	5.76486200
H	18.06445200	4.16832200	5.81842400
H	18.62657100	3.06446400	4.53761400
C	17.00343900	0.85533000	2.37496100
C	17.57159800	-0.54051600	2.07443100
H	18.60057800	-0.60806700	2.44513800
H	17.56828500	-0.72269100	0.99367400
H	16.96751700	-1.30950100	2.56773400
C	15.54575100	0.95296100	1.91317200
H	14.92770100	0.23149200	2.45984600
H	15.48270800	0.73226300	0.84175800
H	15.16439800	1.96590100	2.07372500
H	12.68692500	-0.79975500	5.94485200
H	13.54088600	-2.34668200	6.11830100
H	13.55230200	-1.50070600	4.54695600
H	11.25374000	2.61701100	-1.84420900
H	11.63163300	4.17581600	-2.62354100
H	10.54339000	4.12752600	-1.20648500

3 (triplet, small-core)

U	12.35179200	4.90902100	4.55239800
K	8.86214400	4.23525800	4.25934500
S	13.45668800	5.63932900	6.64283400
Si	10.47604600	2.39808100	6.77274000
Si	9.67070800	7.69534000	4.50595400
Si	10.71231700	3.19387600	1.44494100
Si	15.71083900	5.29993400	2.80564800
O	11.15066300	3.30900100	5.61579700
O	11.01625300	0.80761100	6.82780500
O	10.49821700	2.89811500	8.35882500
O	8.81323900	2.43082800	6.36681200
O	10.73771300	6.47928900	4.43771400
O	9.78442700	8.73883100	5.80220100
O	8.15544300	6.90808600	4.46966500
O	9.62269700	8.71445400	3.17311800
O	11.31794100	3.97480900	2.72293700
O	9.06491600	2.96153700	1.82684200
O	10.85444100	3.98952100	-0.02631500
O	11.25511700	1.64229600	1.12584200
O	14.21007000	5.05517500	3.37728300
O	15.55549600	5.99437600	1.28083000
O	16.58276300	3.87939600	2.56244400
O	16.72922200	6.21721600	3.76942000

C	11.46438600	-0.01761500	5.73294400
C	10.47691500	0.04014000	4.56010900
H	10.45762800	1.04453600	4.12762500
H	9.47247000	-0.22198400	4.91099400
H	10.78006500	-0.66141100	3.77476600
C	12.86302900	0.43201200	5.28732800
H	12.81728600	1.46683100	4.93678500
H	13.22769700	-0.20806600	4.47503200
H	13.55990200	0.37813200	6.13080900
C	11.52299500	-1.44370300	6.30538000
H	12.20268800	-1.46509600	7.16460600
H	11.88056700	-2.14667800	5.54368800
H	10.52421200	-1.74803400	6.63895900
C	11.58067300	2.93596000	9.33061000
C	12.93132200	2.53027900	8.72831000
H	13.21551600	3.24276000	7.94228200
H	12.87549600	1.51574000	8.32249800
H	13.69725600	2.56268400	9.51356700
C	11.17698700	1.96903200	10.45535500
H	10.19922600	2.25806300	10.85929600
H	11.91946100	1.99734500	11.26197300
H	11.11545700	0.94894400	10.05872100
C	11.65038900	4.37792400	9.84844200
H	10.66788500	4.68304500	10.22904400
H	11.95667100	5.03736400	9.02811900
H	12.38839700	4.45080400	10.65698400
C	7.70355900	2.03917900	7.20382600
C	8.01696000	0.77063200	8.01412200
H	8.31312500	-0.03950700	7.34047200
H	7.12767000	0.46634100	8.57997500
H	8.83810100	0.96602300	8.70861700
C	7.34364100	3.20265300	8.14143700
H	7.09410000	4.09175000	7.54947400
H	8.20592100	3.43203700	8.77266700
H	6.48116300	2.93814400	8.76581700
C	6.53597200	1.75596800	6.24150600
H	6.81697500	0.95805400	5.54493600
H	6.29235200	2.66011600	5.66875400
H	5.64413400	1.45023000	6.80099500
C	10.01853800	8.48989500	7.21410400
C	9.67580400	7.04775200	7.60630100
H	10.39008700	6.35289700	7.15227500
H	8.65553800	6.80287700	7.28727800
H	9.74485200	6.93608900	8.69434000
C	11.49028800	8.79440700	7.51625100
H	12.13840700	8.06950600	7.00861600
H	11.67480100	8.71268900	8.59449800
H	11.73075800	9.81329200	7.18861100
C	9.10545600	9.48019900	7.95628500
H	8.05443800	9.25917700	7.73777700
H	9.32757800	10.50211400	7.62800500
H	9.27019200	9.40569600	9.03753200
C	6.86130500	7.48569800	4.18550300
C	6.71345300	8.89794800	4.77192600
H	6.89030900	8.87768500	5.85086000
H	5.69960400	9.26951000	4.57816100
H	7.43897800	9.57336700	4.31109900
C	6.65535600	7.51626100	2.66286800
H	6.72783400	6.50055600	2.25619600
H	7.43608000	8.13275000	2.20941900
H	5.66729700	7.92639100	2.41951100
C	5.83168700	6.54800200	4.84023800
H	6.01676600	6.48903500	5.91858200
H	5.90947800	5.54139000	4.40950100
H	4.81430400	6.91821200	4.66834300
C	10.51540000	9.81858100	2.88855800
C	11.95151100	9.50181800	3.32455300
H	12.00388100	9.37395800	4.41048500
H	12.61974000	10.32096600	3.03290100
H	12.29304600	8.57927500	2.84569000
C	9.99378400	11.07672200	3.60103900
H	8.97444500	11.29713300	3.26115700
H	10.63792100	11.93485700	3.37290300
H	9.98193500	10.90113700	4.68038100
C	10.45840200	10.01277200	1.36539000
H	9.41946600	10.17153200	1.05332400
H	10.84743900	9.11955400	0.86579600
H	11.05986300	10.88070200	1.07023200

C	8.08969700	2.16866700	1.11375100
C	6.71790000	2.75646600	1.49083000
H	6.56923100	2.69288800	2.57638800
H	5.91370700	2.19633600	0.99958100
H	6.66540500	3.80655600	1.18026400
C	8.27655100	2.23803700	-0.41023300
H	8.24277100	3.27694900	-0.75058700
H	7.47561900	1.66962900	-0.89937600
H	9.24313200	1.81122200	-0.69115100
C	8.18808800	0.71289200	1.59506300
H	8.02424800	0.66932300	2.67756000
H	9.19008200	0.33362400	1.37760700
H	7.43608500	0.09197500	1.09236900
C	10.79468700	5.41565400	-0.26368000
C	12.14917800	6.04240100	0.07979100
H	12.93557400	5.60176100	-0.54136300
H	12.40264500	5.85649300	1.12630900
H	12.12342000	7.12521200	-0.09481200
C	9.66957400	6.06534300	0.55248700
H	8.72729400	5.53516700	0.37384800
H	9.55064300	7.11452900	0.26179900
H	9.90851100	6.03892500	1.61989800
C	10.50547300	5.55912500	-1.76720700
H	9.53891300	5.10044400	-2.00589700
H	11.28850700	5.05014300	-2.34046100
H	10.48290600	6.61809800	-2.05019700
C	12.51961900	1.25911100	0.53158000
C	12.38396400	1.31445700	-0.99856400
H	12.13375400	2.33461000	-1.30270700
H	11.58408400	0.63740600	-1.32273100
H	13.32471200	1.00785500	-1.47219900
C	12.76204700	-0.18879400	0.98564900
H	12.85423600	-0.22164700	2.07611200
H	13.68180500	-0.57933500	0.53420200
H	11.91649000	-0.81693300	0.68132600
C	13.66006000	2.16154700	1.01161000
H	13.72842200	2.14731300	2.10250100
H	13.50339400	3.19831600	0.69879200
H	14.61318400	1.81599700	0.59643400
C	16.48965200	6.00229600	0.18615600
C	16.09698500	7.21113100	-0.68107400
H	15.05910500	7.10273200	-1.01264600
H	16.75323700	7.28412300	-1.55697800
H	16.18164000	8.12863700	-0.08758200
C	16.32050700	4.69986500	-0.61360100
H	16.58412100	3.85163200	0.02511200
H	16.96244300	4.70823700	-1.50381600
H	15.27521000	4.59795900	-0.92391000
C	17.94855400	6.14920700	0.65038200
H	18.21844000	5.31054200	1.29790400
H	18.07394300	7.08064700	1.20940800
H	18.61213300	6.16190800	-0.22374100
C	17.21699000	3.06769100	3.57579400
C	17.35459000	1.66912700	2.95144100
H	17.96299000	1.72954700	2.04120900
H	16.36240200	1.28729100	2.68931600
H	17.83189400	0.98117300	3.65977300
C	18.61021300	3.64443400	3.88006000
H	18.50018200	4.65545300	4.28130400
H	19.19827000	3.68888700	2.95531000
H	19.13336900	3.00979100	4.60632600
C	16.37524700	2.99114900	4.85804400
H	16.21004000	3.98619000	5.28345500
H	16.89031600	2.37024800	5.60163400
H	15.39768600	2.54772500	4.64387800
C	16.56248400	7.51338300	4.38582200
C	17.69887500	8.40075000	3.84853600
H	18.66497900	7.92442200	4.05158800
H	17.67261700	9.38412300	4.33368500
H	17.58859200	8.53220100	2.76662900
C	16.71112500	7.31852200	5.90064400
H	15.88740500	6.69465000	6.26896500
H	16.67670000	8.28936300	6.41141300
H	17.67089200	6.83345200	6.11630700
C	15.20097000	8.14208900	4.06266300
H	14.40533400	7.55255400	4.53070500
H	15.05049100	8.18024200	2.97735100
H	15.16297000	9.16106100	4.46741500

3 (singlet, large-core)

U	12.37203600	5.09672900	4.58983400
K	8.89501900	4.17801500	4.20225600
S	13.40289900	6.09565500	6.70854700
Si	10.72723800	2.43565200	6.77325900
Si	9.48866500	7.66114400	4.56928500
Si	10.64296900	3.31164300	1.37520700
Si	15.73218800	5.38280300	2.82488100
O	11.42253900	3.25961100	5.56391400
O	11.18993700	0.82236700	6.86261000
O	10.86113400	3.00447200	8.32817900
O	9.05650900	2.54796600	6.43394800
O	10.55222300	6.43896200	4.50071600
O	9.55999200	8.66706000	5.89461700
O	7.99080500	6.85686300	4.45274700
O	9.51292600	8.71112700	3.26209700
O	11.21618300	4.07633900	2.66287300
O	8.95985800	3.15210700	1.66763900
O	10.89409800	4.05139700	-0.11627400
O	11.11636100	1.72005200	1.10158300
O	14.17352400	5.32105500	3.27873300
O	15.76328700	6.03486700	1.27772700
O	16.45977700	3.86888700	2.70201300
O	16.74466800	6.23021100	3.85488500
C	11.48481300	-0.07530900	5.77125300
C	10.48181500	0.08678900	4.61982900
H	10.59478000	1.06877100	4.15005200
H	9.46010200	-0.02025100	5.00022200
H	10.66154300	-0.67811100	3.85573900
C	12.91414300	0.18700100	5.27740800
H	12.98140800	1.21139000	4.90075700
H	13.17614000	-0.51134100	4.47344100
H	13.62214600	0.06579000	6.10470000
C	11.37965300	-1.48695000	6.37119300
H	12.07197700	-1.57772200	7.21575200
H	11.62851800	-2.24221900	5.61634300
H	10.35941500	-1.65967300	6.73247500
C	11.98639300	2.91419200	9.24692600
C	13.31843400	2.70199700	8.51604700
H	13.50218600	3.54288700	7.83486400
H	13.30240300	1.75706900	7.96276600
H	14.13115700	2.66812100	9.25263800
C	11.69390800	1.74476800	10.19926100
H	10.73844100	1.91319500	10.71099000
H	12.48925000	1.65874500	10.94972600
H	11.63615300	0.81419300	9.62380200
C	12.01475200	4.25106000	9.99875300
H	11.04444700	4.42705200	10.47917600
H	12.22790400	5.05462800	9.28398600
H	12.79861100	4.23451700	10.76598200
C	7.96455000	2.17923400	7.30748100
C	8.25802800	0.88488500	8.08307000
H	8.48345900	0.07100400	7.38707000
H	7.38258100	0.61437200	8.68634100
H	9.11847400	1.02816300	8.74194600
C	7.68562400	3.33570700	8.27964300
H	7.45371000	4.24673400	7.71575600
H	8.57772000	3.51884000	8.88395900
H	6.83530200	3.09155400	8.92849300
C	6.75062000	1.95996700	6.38644800
H	6.97654300	1.17546700	5.65513100
H	6.50781200	2.88910200	5.85479500
H	5.87428500	1.66288100	6.97411300
C	9.69932800	8.41578200	7.31896800
C	9.58243800	6.92684600	7.65886600
H	10.43553800	6.37745500	7.24614200
H	8.64065100	6.52373500	7.26672100
H	9.59399700	6.79821900	8.74739200
C	11.06795200	8.95695900	7.75007400
H	11.86591300	8.36101200	7.29003300
H	11.17194000	8.88378500	8.83972900
H	11.15920800	10.00863300	7.45180800
C	8.57196700	9.21299500	7.99552700
H	7.59667500	8.82568300	7.67978600
H	8.64347800	10.26893700	7.71060600
H	8.65391500	9.12751300	9.08549900
C	6.71376500	7.41169100	4.06231600

C	6.49579400	8.82067900	4.63396400
H	6.57414100	8.80206200	5.72453300
H	5.49898400	9.18065300	4.35044400
H	7.25088000	9.50556600	4.23882200
C	6.63409400	7.43941900	2.52777900
H	6.77028600	6.42708900	2.13025600
H	7.43076900	8.07889400	2.13829200
H	5.65802700	7.82104300	2.20345200
C	5.65287400	6.45345000	4.63050800
H	5.74795400	6.39912000	5.72077100
H	5.79008000	5.44942000	4.20965200
H	4.64618900	6.80228700	4.37269100
C	10.41803900	9.82453700	3.05518700
C	11.83418500	9.49277600	3.54363900
H	11.84202100	9.32379100	4.62527500
H	12.51139700	10.32383500	3.31323900
H	12.20270400	8.59318000	3.04063200
C	9.86582600	11.05956200	3.78425700
H	8.86352500	11.29368000	3.40528800
H	10.52101400	11.92256700	3.61336100
H	9.80472800	10.84910700	4.85545600
C	10.42815800	10.06733700	1.53820500
H	9.40710400	10.26023600	1.18883900
H	10.81705300	9.18085600	1.02709100
H	11.06109900	10.92929700	1.29625900
C	8.00022200	2.34310800	0.95778200
C	6.62575800	2.97297900	1.24843800
H	6.42801800	2.96145300	2.32809300
H	5.83119700	2.41009300	0.74450100
H	6.61269300	4.01043700	0.89579100
C	8.25044700	2.33926000	-0.55857500
H	8.24329200	3.36297200	-0.94440700
H	7.46580200	1.75710700	-1.05794100
H	9.22442000	1.89358300	-0.77595600
C	8.04606000	0.90858200	1.50928000
H	7.84542900	0.92079000	2.58732200
H	9.04386600	0.49424100	1.34400100
H	7.29287800	0.28299700	1.01402300
C	10.90002200	5.46719400	-0.40399000
C	12.27938900	6.04352400	-0.06200400
H	13.05107300	5.54016000	-0.65499400
H	12.50963200	5.88358500	0.99475000
H	12.31124400	7.11852400	-0.27962700
C	9.79346300	6.20062200	0.36645900
H	8.82552500	5.72785700	0.16630900
H	9.74981600	7.24895900	0.05080400
H	9.99206900	6.17504900	1.44214800
C	10.64082000	5.57210800	-1.91675600
H	9.66080100	5.14307000	-2.15541200
H	11.41189000	5.01378300	-2.45967900
H	10.66419900	6.62091900	-2.23563400
C	12.34832700	1.26250800	0.49953900
C	12.19372700	1.27291000	-1.03040500
H	12.00513000	2.29602700	-1.36705700
H	11.34654400	0.63846700	-1.31852200
H	13.10458100	0.88823000	-1.50579800
C	12.53892500	-0.18022200	0.99499900
H	12.63384000	-0.18423400	2.08558600
H	13.44174200	-0.61773800	0.55225800
H	11.66931900	-0.78500600	0.71177300
C	13.53892200	2.12729000	0.92935300
H	13.62689600	2.13907000	2.01944500
H	13.41521500	3.15913300	0.58710800
H	14.46683300	1.72713900	0.50422500
C	16.71206800	5.84341300	0.21461700
C	16.52899500	7.05423200	-0.71614900
H	15.48627800	7.10194900	-1.04866700
H	17.18687600	6.97083100	-1.58977400
H	16.76613300	7.97468700	-0.17032000
C	16.36157100	4.54516400	-0.53071200
H	16.45947500	3.70030400	0.15757800
H	17.02964200	4.40304500	-1.38972800
H	15.32580200	4.59691500	-0.88329800
C	18.16241700	5.78685700	0.72318300
H	18.28358300	4.93237700	1.39458500
H	18.40495700	6.70278100	1.26978100
H	18.84779300	5.68238200	-0.12758300
C	16.92759400	3.03301800	3.78296800

C	17.02113400	1.61731900	3.18988100
H	17.70162200	1.62495900	2.33036600
H	16.03081800	1.29355300	2.85284300
H	17.39706300	0.91286300	3.94150100
C	18.32037900	3.50799500	4.22834900
H	18.25097700	4.53600300	4.59295100
H	19.00760800	3.47751800	3.37414000
H	18.70682600	2.85572700	5.02133900
C	15.95075000	3.04301400	4.96782300
H	15.84748600	4.04770000	5.39276000
H	16.30893000	2.36643400	5.75307800
H	14.96576600	2.69580600	4.63555200
C	16.69635500	7.59931400	4.32039400
C	17.84078900	8.35304600	3.62246500
H	18.78840900	7.83289000	3.80457600
H	17.91089600	9.37681900	4.00993200
H	17.65546300	8.39065200	2.54317700
C	16.93036800	7.54768900	5.83681100
H	16.10006500	7.00886600	6.30950700
H	16.97750700	8.56401100	6.24779200
H	17.87579600	7.03205400	6.04579700
C	15.35282800	8.27663500	4.02124200
H	14.55843200	7.77268100	4.58284200
H	15.13838400	8.24013100	2.94639800
H	15.39704000	9.32526500	4.34167900

5 (triplet, small-core)

U	1.01940200	9.19326800	11.29990900
Si	3.06079700	9.68450500	8.18052100
Si	3.97163100	8.55504700	13.53156300
Si	-1.11996900	6.46071700	9.82006900
Si	-0.81956900	12.44533600	10.86826900
H	0.98814100	8.47915100	14.33252400
S	-0.14656500	8.98616400	13.81138900
O	3.27587400	8.53233200	15.05644000
O	4.94225900	7.19366100	13.37419300
O	-0.28106400	10.90971500	11.05506900
O	-1.07251400	13.26211500	12.30890900
O	-2.21859100	12.34856200	9.95314800
O	0.23812100	13.43210400	10.02358800
O	-0.02480800	7.46868500	10.50097900
O	-1.68720800	5.27920400	10.86886400
O	-2.49822500	7.19113700	9.22336400
O	-0.36322800	5.79018800	8.48368700
O	2.80627900	8.59648900	12.38022700
O	4.99882800	9.87145500	13.46724000
O	2.21837400	9.74378200	9.58304500
O	4.26558400	8.52454300	8.11857600
O	3.88171700	11.13962000	8.02907700
O	2.09111300	9.37926200	6.85049000
C	4.17706800	7.08451000	8.03525000
C	5.58637300	6.57590600	8.38058000
H	6.31522200	7.00554500	7.68329000
H	5.62197200	5.48220500	8.31036300
H	5.84657900	6.88092600	9.39933400
C	3.80895900	6.67900400	6.59949300
H	2.83341100	7.09928400	6.33974700
H	3.77663100	5.58576300	6.51500600
H	4.55950000	7.06955100	5.90178400
C	3.16005900	6.53233100	9.03945200
H	3.39856900	6.89713000	10.04468300
H	3.19660000	5.43612100	9.03837800
H	2.13992200	6.83325600	8.78234500
C	4.98876600	11.43656800	7.14779200
C	6.29643200	11.04844600	7.85528700
H	6.36509700	11.57969500	8.81072500
H	7.15933400	11.31389800	7.23129100
H	6.29539500	9.97188300	8.04714200
C	4.88269400	10.70778000	5.79671500
H	4.90913600	9.62568000	5.95172300
H	5.72520700	11.00236400	5.15835500
H	3.94542700	10.96946500	5.29669000
C	4.94674300	12.95638400	6.92178400
H	4.00362200	13.23305700	6.43821800
H	5.78477400	13.26873500	6.28694000
H	5.01088400	13.47276600	7.88520400
C	0.87984600	10.04610000	6.42230500
C	-0.29253200	9.65368300	7.32834600

H	-0.34513500	8.56637200	7.42785400
H	-1.23340500	10.01868500	6.89880300
H	-0.17879200	10.09210700	8.32342500
C	1.05725000	11.57126000	6.40740300
H	1.21708400	11.94996700	7.42196300
H	0.15244200	12.03944400	6.00239100
H	1.91327700	11.84763700	5.78317200
C	0.63835800	9.53004800	4.99371600
H	1.50063900	9.77305300	4.36221300
H	-0.26362400	9.98751300	4.57064300
H	0.51076000	8.44230900	5.01703600
C	3.28498600	9.52817700	16.10964000
C	2.28263900	9.01437800	17.15468100
H	1.28619200	8.94815500	16.70488100
H	2.24723100	9.69834000	18.01111500
H	2.58684700	8.01944500	17.50034000
C	4.69309400	9.61437500	16.71850300
H	4.99656600	8.62946500	17.09182600
H	4.69872500	10.32915900	17.55086300
H	5.40127000	9.94263200	15.95236300
C	4.67390300	5.85099700	13.83278600
C	5.22903300	5.71298700	15.25918500
H	4.70253400	6.41123300	15.91679400
H	6.29777100	5.95668500	15.26144000
H	5.09154600	4.68870000	15.62712100
C	6.15881100	10.06403700	12.62334100
C	7.38589400	9.51408400	13.36787000
H	7.48707900	10.01788500	14.33632700
H	8.29541300	9.68370400	12.77838500
H	7.25346400	8.44061700	13.53488100
C	5.99364600	9.37029500	11.26725600
H	5.91366600	8.28760900	11.40826700
H	6.86313300	9.58365500	10.63483500
H	5.09472700	9.72704700	10.75731300
C	6.28133900	11.58339500	12.43414900
H	5.39106500	11.96010900	11.91967600
H	7.17019300	11.82148900	11.83775200
H	6.36114800	12.07211800	13.41213500
C	-0.93472000	4.44946200	11.78308700
C	0.48426500	4.17612400	11.26207000
H	0.44025100	3.71245000	10.27121900
H	1.01079600	3.50706700	11.95289700
H	1.04382500	5.11361100	11.18386200
C	-0.89377800	5.14200800	13.15073500
H	-0.40876000	6.12009300	13.07422800
H	-0.34851600	4.52418900	13.87518100
H	-1.91582000	5.29895500	13.51447400
C	-1.72029500	3.13077000	11.88303700
H	-2.74258000	3.34023100	12.21734700
H	-1.23864300	2.45433300	12.59928900
H	-1.76267900	2.64661900	10.90114400
C	-3.65271500	7.66912200	9.95580000
C	-3.25523200	8.26585200	11.31105500
H	-2.78694900	7.50796500	11.94717100
H	-4.14638500	8.65182700	11.82082000
H	-2.55161900	9.09138800	11.16756500
C	-4.63022700	6.49742700	10.13966100
H	-4.91238900	6.09589200	9.15873900
H	-5.53458500	6.83755200	10.65934300
H	-4.14470000	5.70993800	10.72359700
C	-4.27728100	8.75285600	9.06501100
H	-3.57079800	9.58159200	8.95093600
H	-5.20160500	9.13003700	9.51882200
H	-4.50420700	8.33206700	8.07840200
C	-0.94014100	5.01396600	7.41328500
C	-1.46908500	5.97067800	6.33361600
H	-2.25219800	6.60234700	6.76313900
H	-1.87319100	5.40207200	5.48656700
H	-0.65376900	6.60999400	5.97955900
C	-2.07090300	4.09199600	7.89875400
H	-1.70784600	3.43117000	8.69145500
H	-2.43063100	3.48352900	7.05950000
H	-2.90057700	4.68671000	8.29211800
C	0.21746900	4.17147200	6.85232300
H	1.03133600	4.83338800	6.53854000
H	-0.12089100	3.57838200	5.99403000
H	0.59381800	3.49817500	7.63102200
C	-1.90991800	12.96017500	13.45058700

C	-0.97999600	12.68771400	14.64055500
H	-0.40337400	11.77576300	14.45414100
H	-1.56779900	12.54784300	15.55628800
H	-0.29929700	13.53653300	14.77610400
C	-2.82756300	11.75638700	13.20013100
H	-3.44603500	11.92842400	12.31299000
H	-3.47628000	11.60266800	14.07106500
H	-2.22834200	10.85305600	13.05418000
C	-2.74491300	14.22456900	13.71501900
H	-2.07764900	15.08249300	13.85673300
H	-3.35798300	14.09218600	14.61458600
H	-3.40029400	14.42490100	12.86092900
C	-2.85495000	13.35766700	9.14301000
C	-4.33521500	12.94927800	9.06847100
H	-4.41545800	11.93573500	8.66260100
H	-4.89178900	13.64340000	8.42717900
H	-4.77027800	12.95932700	10.07450100
C	-2.73225900	14.76455900	9.74888800
H	-3.15632700	14.77683300	10.75702800
H	-3.27600700	15.48236100	9.12196000
H	-1.68007200	15.05678200	9.80385300
C	-2.22288400	13.32548300	7.74340900
H	-1.16067300	13.57525400	7.82276000
H	-2.72251300	14.04321500	7.08048400
H	-2.31890200	12.31812200	7.32447800
C	1.42101400	14.09980800	10.52887200
C	2.19707200	13.20859500	11.50995300
H	1.59015700	12.99139800	12.39546100
H	3.11214800	13.72236400	11.82907500
H	2.47587200	12.27167200	11.01619800
C	2.28526700	14.38982600	9.29457600
H	2.59426500	13.44874700	8.82948400
H	3.17870200	14.95606400	9.58546900
H	1.70721400	14.97881800	8.57266100
C	1.00139400	15.41493200	11.20526100
H	0.47510200	16.04890900	10.48159900
H	1.88747500	15.95061600	11.56797500
H	0.33331600	15.19544200	12.04245200
C	2.83332900	10.89316100	15.57185900
H	3.54969300	11.25674300	14.82891700
H	2.76893200	11.61419600	16.39637100
H	1.84739100	10.79167400	15.10545900
C	3.17395400	5.52847100	13.80420200
H	2.77856200	5.67870300	12.79596700
H	2.63117000	6.18151800	14.49537900
H	3.01298600	4.48447700	14.09998500
C	5.43533200	4.92609500	12.87044100
H	5.32116300	3.87909000	13.17582400
H	6.49946300	5.18874400	12.87402100
H	5.04372800	5.05112400	11.85518400
